# Multilocus Sequence Typing Reveals Extensive Genetic Diversity of the Emerging Fungal Pathogen *Scedosporium aurantiacum*

**DOI:** 10.3389/fcimb.2021.761596

**Published:** 2021-12-27

**Authors:** Azian Harun, Alex Kan, Katharina Schwabenbauer, Felix Gilgado, Haybrig Perdomo, Carolina Firacative, Heidemarie Losert, Sarimah Abdullah, Sandrine Giraud, Josef Kaltseis, Mark Fraser, Walter Buzina, Michaela Lackner, Christopher C. Blyth, Ian Arthur, Johannes Rainer, José F. Cano Lira, Josep Guarro Artigas, Kathrin Tintelnot, Monica A. Slavin, Christopher H. Heath, Jean-Philippe Bouchara, Sharon C. A. Chen, Wieland Meyer

**Affiliations:** ^1^ Molecular Mycology Research Laboratory, Centre for Infectious Diseases and Microbiology, Faculty of Medicine and Health, Sydney Medical School, Westmead Clinical School, Sydney Institute for Infectious Diseases, Westmead Hospital-Research and Education Network, Westmead Institute for Medical Research, University of Sydney, Sydney, NSW, Australia; ^2^ School of Medical Sciences, Universiti Sains Malaysia, Kota Bharu, Malaysia; ^3^ Unitat de Microbiologia, Facultat de Medicina i Ciencies de la Salut, Universitat Rovira i Virgili, Reus, Spain; ^4^ FG 16 Mycology, Robert Koch Institute, Berlin, Germany; ^5^ UNIV Angers, Université de Bretagne Occidentale, Centre Hospitalier Universitaire (CHU) d’Angers, Groupe d’Etude des Interactions Hôte-Pathogène (GEIHP), EA3142, Structure Fédérative de Recherche “Interactions Cellulaires et Applications Thérapeutiques (SFR ICAT), Angers, France; ^6^ Institute of Hygiene and Microbiology, Medical University Innsbruck, Innsbruck, Austria; ^7^ UK National Mycology Reference Laboratory, National Infection Service, Public Health England South-West, Bristol, United Kingdom; ^8^ Institute of Hygiene, Microbiology and Environmental Medicine, Medical University, Graz, Austria; ^9^ Telethon Kids Institute and Medical School, University of Western Australia, Perth, WA, Australia; ^10^ Mycology Laboratory, Division of Microbiology and Infectious Diseases, PathWest Laboratory Medicine Western Australia, Perth, WA, Australia; ^11^ Institute of Microbiology, Leopold Franzens University Innsbruck, Innsbruck, Austria; ^12^ Peter MacCallum Cancer Centre and Sir Peter MacCallum Department of Oncology, Melbourne, VIC, Australia; ^13^ Department of Microbiology, PathWest Laboratory Medicine, Fiona Stanley Hospital, Murdoch; & Infectious Diseases Department, Fiona Stanley Hospital, Murdoch; Department of Microbiology & Infectious Diseases, Royal Perth Hospital, Perth; & the University of Western Australia, Perth, WA, Australia; ^14^ Center for Infectious Diseases and Microbiology Laboratory Services, Institute of Clinical Pathology and Medical Research, New South Wales Health Pathology, Sydney, NSW, Australia

**Keywords:** *Scedosporium aurantiacum*, MLST (multilocus sequence typing), genotyping, geographical origins, ecological context, clinical association

## Abstract

*Scedosporium* spp. are the second most prevalent filamentous fungi after *Aspergillus* spp. recovered from cystic fibrosis (CF) patients in various regions of the world. Although invasive infection is uncommon prior to lung transplantation, fungal colonization may be a risk factor for invasive disease with attendant high mortality post-transplantation. Abundant in the environment, *Scedosporium aurantiacum* has emerged as an important fungal pathogen in a range of clinical settings. To investigate the population genetic structure of *S. aurantiacum*, a MultiLocus Sequence Typing (MLST) scheme was developed, screening 24 genetic loci for polymorphisms on a tester strain set. The six most polymorphic loci were selected to form the *S. aurantiacum* MLST scheme: actin (*ACT*), calmodulin (*CAL*), elongation factor-1α (*EF1*α), RNA polymerase subunit II (*RPB2*), manganese superoxide dismutase (*SOD2*), and β-tubulin (*TUB*). Among 188 global clinical, veterinary, and environmental strains, 5 to 18 variable sites per locus were revealed, resulting in 8 to 23 alleles per locus. MLST analysis observed a markedly high genetic diversity, reflected by 159 unique sequence types. Network analysis revealed a separation between Australian and non-Australian strains. Phylogenetic analysis showed two major clusters, indicating correlation with geographic origin. Linkage disequilibrium analysis revealed evidence of recombination. There was no clustering according to the source of the strains: clinical, veterinary, or environmental. The high diversity, especially amongst the Australian strains, suggests that *S. aurantiacum* may have originated within the Australian continent and was subsequently dispersed to other regions, as shown by the close phylogenetic relationships between some of the Australian sequence types and those found in other parts of the world. The MLST data are accessible at http://mlst.mycologylab.org. This is a joined publication of the ISHAM/ECMM working groups on “*Scedosporium/Pseudallescheria* Infections” and “Fungal Respiratory Infections in Cystic Fibrosis”.

## Introduction

Fungi of the genera *Scedosporium* and *Lomentospora* are increasingly encountered as causes of invasive fungal infections (Cortez et al., 2008; Heath et al., 2009; Nakamura et al., 2013; Lass-Flörl and Cuenca-Estrella, 2017; Kondo et al., 2018; Ramirez-Garcia et al., 2018; Chen et al., 2021; Mizusawa et al., 2021). Moreover, these fungi are frequently associated with airway colonization, particularly in the context of abnormal airway function in chronic respiratory disease (Cortez et al., 2008; Pihet et al., 2009; Blyth et al., 2010; Zouhair et al., 2013; Schwarz et al., 2018). Infections due to *Scedosporium/Lomentospora* spp. are noteworthy due to their inherent resistance to most available antifungal agents (Gilgado et al., 2006; Troke et al., 2008; Lackner et al., 2014a; Rivero-Menendez et al., 2020). Recent taxonomic reassignments within these genera and the identification of new *Scedosporium* species/species complex (Guarro et al., 1999; Gilgado et al., 2005; Gilgado et al., 2008; Lackner et al., 2014b; Chen et al., 2016; Chen et al., 2021) have raised the need to gain better insight into the epidemiology of clinically relevant species.

*Scedosporium aurantiacum* has emerged as a pathogen with a relatively high prevalence in Australia and is often associated with chronic lung disease (Delhaes et al., 2008; Heath et al., 2009). *In vivo* experiments in mice showed that *S. aurantiacum* is as virulent as *Lomentospora prolificans* (former *Scedosporium prolificans*) (Harun et al., 2010b), and more virulent than the other members of the genus (Gilgado et al., 2009; Rodriguez et al., 2010). Further, *S. aurantiacum* was found to be highly abundant in the Australian environment (Harun et al., 2010a), although a specific association between its occurrence in the environment and the relatively high clinical incidence has not yet been explored. Given the emerging nature and the poor clinical outcomes generally associated with *Scedosporium/Lomentospora* spp. infections, a better understanding of the epidemiology and transmission is necessary to ensure effective therapeutic and preventative measures. Therefore, an investigation of the population genetic structure of this pathogen is crucial to enable a correlation between the observed genotype, source of isolation (clinical and environmental), virulence, antifungal susceptibility, and clinical outcome.

Several molecular typing techniques have been applied to isolates of the genera *Scedosporium* and *Lomentospora*, to detect genetic variation over time, to discriminate between strains and to identify possible sources of infection. Among those methods are: Random Amplified Polymorphic DNA (RAPD) analysis (Zouhair et al., 2001; Defontaine et al., 2002), Multi-Locus isoEnzyme Electrophoresis (MLEE) (Zouhair et al., 2001), Amplified Fragment Length Polymorphism (AFLP) (Delhaes et al., 2008), PCR fingerprinting (Rainer et al., 2000; Delhaes et al., 2008) and MultiLocus Sequence Typing (MLST) (Bernhard et al., 2013). However, many of these studies were conducted prior to the taxonomical resolution of the *Scedosporium boydii* species complex, with few data, if any, describing the genetic diversity within *S. aurantiacum*. MLST has been successfully applied to study the genetic diversity of medically important fungi, including *Candida albicans* (Bougnoux et al., 2002), *Candida glabrata* (Dodgson et al., 2003; Lott et al., 2010), *Candida tropicalis* (Tavanti et al., 2005), *Candida krusei* (Jacobsen et al., 2007), *Cryptococcus gattii* (Feng et al., 2008; Meyer et al., 2009; Carriconde et al., 2011), *Cryptococcus neoformans* var. *grubii* (Litvinseva et al., 2006), *Aspergillus fumigatus* (Bain et al., 2007), and *Fusarium solani* species complex (Debourgogne et al., 2010), but has only been applied to *Scedosporium apiospermum* and *S. boydii* (formerly *Pseudallescheria boydii*) within the genus *Scedosporium* (Bernhard et al., 2013). It has a strong advantage over other typing techniques, as it provides unambiguous data, allowing for inter-laboratory data comparisons, construction of large international, internet-accessible databases (www.mlst.net or http://mlst.mycologylab.org) (Maiden et al., 1998; Odds and Jacobsen, 2008; Carriconde et al., 2011), and is only exceeded in its discriminatory power by whole genome sequencing, which is not yet feasible in most clinical mycology laboratories.

The current study describes the development of an MLST scheme specific for *S. aurantiacum* and its application to a global set of *S. aurantiacum* isolates. Partial sequences from six genetic loci, including: actin (*ACT*), calmodulin (*CAL*), elongation factor 1 alpha (*EF1α*), RNA polymerase II subunit (*RPB2*), superoxide dismutase (*SOD2*) and beta tubulin (*TUB*), were obtained from a population of 188 *S. aurantiacum* strains. Genetic relatedness between strains from different geographical origins, ecological contexts, and clinical associations were examined.

## Material And Methods

### Isolates

During the development phase, 12 *S. aurantiacum* strains were selected as tester strains from the Molecular Mycology Research Laboratory Culture Collection at Sydney Medical School - Westmead Hospital (as indicated by the strain numbers in bold and italics in **Supplementary Table S2**). These strains were selected to represent diverse geographic regions, had known clinical associations, and known genetic characteristics (identical and diverse genotypes) as established by PCR fingerprinting and AFLP analysis (Delhaes et al., 2008). In the application phase, the developed scheme was applied to a total of 188 strains that comprised 106 clinical, 1 veterinary and 81 environmental strains. Details of these strains are provided in **Supplementary Table S2**. Most of strains originated from Australia (n = 84), followed by France (n = 48), Austria (n = 15), Germany (n = 13), Spain (n = 5), New Zealand (n = 4), the UK (n = 3), Nepal (n = 2), Thailand (n = 2), Ireland (n = 1), and the USA (n = 1). All isolates were grown on Sabouraud dextrose agar (Oxoid, Hampshire, UK) and incubated at 30°C for 5 to 7 days before DNA extraction.

### DNA Extraction

Extraction of genomic DNA was performed according to a previously published protocol (Ferrer et al., 2001), with minor modification. The mycelia from 5-day-old cultures were harvested and placed in 1.5 ml tubes. After washing in deionized water, mycelia were frozen in liquid nitrogen. Using a sterile miniature pestle, frozen mycelia were finely ground to disrupt the fungal cell walls; 500 µl of SDS lysis buffer and 5 µl of 2-mercaptoethanol were then added and the mixture was mixed vigorously by vortexing. After incubation at 65°C for 1 hour, with 2-3 times vortexing in between, 500 µl of phenol:chloroform:isoamyl alcohol (25:24:1) (Sigma, St. Louis, USA) were added. The tubes were flipped for 2 minutes to ensure thorough mixing, followed by centrifugation at 14,000 rpm for 15 minutes. DNA from the aqueous phase was transferred to a fresh 1.5 ml tube. An equal amount of isopropanol (Merck, Kilsyth, Australia) was then added to precipitate the DNA. The tubes were then incubated at - 20°C for a minimum of 1 hour but usually overnight to increase DNA yield. The precipitated DNA was pelleted by centrifugation at 14,000 rpm for 15 minutes. After washing with 500 µl 70% ethanol (Merck) and centrifugation, the DNA pellet was dried at room temperature and reconstituted in 100 µl of sterile distilled water. DNA concentration was determined spectrophotometrically.

### Selection of Candidate Loci

In the preliminary development stage, 24 gene loci, namely *AAT, ACT, ANXC4, ATP6, BGT, BT2, EF1a, CAL, CAT, CHS, D1D2, FKS, LIP, GLN, IGS, MDH1, mtSSU, MP1, RPB1, RPB2, SOD2, TUB, VPS13* and *ZRF*, were amplified from the 12 tester strains (see above) to identify the most polymorphic loci. These loci had been previously utilized in either phylogenetic and/or genotyping studies of other Scedosporium species and/or other fungi, such as Candida, Aspergillus and Penicillium species (O’Donnell et al., 1998; Liu et al., 1999; Bougnoux et al., 2002; Cruse et al., 2002; Dodgson et al., 2003; Gilgado et al., 2005; Bain et al., 2007; Hoffman et al., 2007). For each genetic locus, the 12 sequences obtained from the tester strains were aligned using BioEdit™ Sequence Alignment Editor (Tom Hall, Carlsbad, USA) to determine the sequence variation. The genetic loci, which demonstrated a high polymorphism and yielded the largest number of sequence types in combination, were selected to form the new S. aurantiacum MLST scheme.

### Primer Design

Twenty-four loci were selected for initial screening of genetic polymorphisms (see above). For the PCR amplification of these loci, primers were used as previously published except for *SOD2* for which primers were specifically designed in the current study (O’Donnell et al., 1998; Liu et al., 1999; Bougnoux et al., 2002; Cruse et al., 2002; Dodgson et al., 2003; Gilgado et al., 2005; Bain et al., 2007; Hoffman et al., 2007) (**Supplementary Table S1**). Partial or full *SOD2* gene sequences were obtained from the GenBank database (http://www.ncbi.nlm.nih.gov). Sequences from as many fungal genera as possible for *SOD2* were downloaded and then aligned using the program BioEdit™ Sequence Alignment Editor. Initial primers for *SOD2* were designed based on the aligned sequences. Following the initial amplification and selection of the six most polymorphic loci, *S. aurantiacum* specific primers were subsequently designed for those loci (**Table 1**) based on the obtained sequences and on the nucleotide sequence of these loci identified *via* BLAST searches in the draft genome of *S. aurantiacum* strain WM 09.24 (available in the DDBJ/EMBL/GenBank under the accession number JUDQ00000000) (Pérez-Bercoff et al., 2015).

**Table 1:** Selected gene loci, primer sequences and annealing temperatures used in the consensus MLST scheme for *Scedosporium aurantiacum* strain typing.

Locus	Coded protein	Sequence 5’-3’	Annealing temperature (°C)	Product length (bp)	Targeted allele length (bp)
*ACT*	Actin	ACT-Sau-F: CTCCTGCTTGGAGATCCACAT	60	998	830
		ACT-Sau-R: TCTCCGCTACCCTATCGAGC			
*CAL*	Calmodulin	CAL-Sau-F: TCTACGTTCGCACGCTAAACT	58	837	689
		CAL-Sau-R: GGAGGAGGGACGCTACTTTTG			
*EF1*	Elongation factor 1-alpha	EF1-Sau-F: CAGCCTGGGAGGTACCAGTAAT	62	859	715
		EF1-Sau-R: AGCGCCTGGATGAGCCAATG			
*RPB2*	RNA polymerase II subunit	RPB2-Sau-F: AGTGTTACGCGGGGACTAAA	62	1214	952
		RPB2-Sau-R: TGATCGTGATCACTTCGGCAA			
*SOD2*	Manganese superoxide dismutase	SOD2-Sau-F: GCCCTACATTAGCGCCAAGA	60	584	437
		SOD2-Sau-R: TTGCGGTTCTCGTACTGGAG			
*TUB*	Beta-tubulin	TUB-Sau-F: CTGTCTCACCCCTCGTACGGTGACCTCAAC	68	676	401
		TUB-Sau-R: GCCCTCGCTAGTGTACCAATGCAAGAAAGC			

### Amplification and DNA Sequencing

PCR amplifications were performed in a total volume of 50 µl. Generally, each reaction mixture contained: 1x PCR buffer (20 mM Tris-HCl, 50 mM KCl), 1.5-2.5 mM MgCl_2_, 100-200 µM of each deoxyribonucleotide triphosphate (dATP, dCTP, dGTP, dTTP) (Invitrogen, Carlsbad, USA), 0.2-0.4 mM each of forward and reverse primer, and 1.25 U of DNA polymerase (Bioline™, London, UK). 20-60 ng of template DNA was added to the reaction mixture. Sterile distilled water in place of DNA was used as a negative control. The primer sequences used in the development phase are listed in **Supplementary Table S1** and for the final consensus MLST scheme in **Table 1**. Initial PCR amplifications were performed in a thermal cycler (Perkin Elmer Cetus, Norwalk, USA) under the following conditions: an initial denaturation at 94°C for 5-10 minutes, followed by 35 cycles of 94°C for 45 seconds at a temperature ranging from 50-60°C for annealing depending on the amplified genetic loci (see **Supplementary Table S1**), followed by an extension step 1 min at 72°C and a denaturation step of 45 seconds at 94°C, with a final extension step at 72°C for 10 min. Optimized annealing temperatures for the primer sets used in the final MLST scheme are as listed in **Table 1**. PCR products were separated on 1.4% agarose gels in Tris-borate-EDTA (TBE) buffer, stained with ethidium bromide (Sigma) and visualized by UV transillumination. The products were purified using PureLink™ PCR Purification Kit (Invitrogen) following the manufacturers protocol and the concentration of purified DNA was measured spectrophotometrically. DNA sequencing was performed by Macrogen Inc., Seoul, Korea (http://www.macrogen.co.kr/eng/sequencing), and the Australian Genome Research Facility (AGRF) Pty. Ltd., St. Lucia, Queensland, Australia (http://www.agrf.org.au). The quality of nucleotide sequences was verified by aligning both forward and reverse strands using the software Sequencher™ 5.4 (Gene Codes Corp., Ann Arbor, USA).

### Sequence Data Analysis

During the development phase, the consensus sequences of the 12 selected strains were aligned for each genetic locus. The genetic loci that were most polymorphic were selected for inclusion in the final *S. aurantiacum* MLST scheme. In the application phase, all obtained sequences from the 188 strains were aligned and analyzed. Sequence alignments were performed using BioEdit™ Sequence Alignment Editor. For each locus, numbers were assigned to designate unique allelic variants, with a single bp difference resulting in a new allele type. These numbers were subsequently combined to yield unique sequence types (see **Supplementary Table S2**). A *S. aurantiacum* MLST database using the BioloMICS software version 21.07.9.324 (BioAware, Hannut, Belgium) was constructed for the six loci at the Molecular Mycology Research Laboratory and can be accessed at http://mlst.mycologylab.org. GenBank accession numbers for all MLST sequences generated in this study are listed in **Supplementary Table S3**. All allele and sequence types can be accessed *via* the specific MLST *S. aurantiacum* website at http://mlst.mycologylab.org. Phylogenetic trees were constructed using the software MEGA version 11 (The Biodesign Institute, Tempe, USA) (Tamura et al., 2021). To further investigate the geographical relationship between genotypes, a goeBURST minimum spanning tree was generated from the aligned concatenated sequences of the strains studied using the PHYLOViZ 2.0 analysis software (http://www.phyloviz.net/).

### Test for Selective Pressure, Variability, and Neutrality

Assessment of the likelihood of selective pressure at each locus was estimated by the ratio of non-synonymous to synonymous nucleotide substitutions (*d_N_/d_S_*) (Nei and Gojobori, 1986). To test for purifying selection the codon-based Z-test using evolutionary pathway by Nei and Gojobori was performed (Nei and Gojobori, 1986) using MEGA version 11 (Tamura et al., 2021). To evaluate the variability of the selected loci, the haplotype diversity (*Hd*), nucleotide diversity (π) and the average number of nucleotide differences (k) were determined using the software DNA Sequence Polymorphism DnaSP version 6.12.03 (Rozas et al., 2017). Testing for neutrality utilizing the Tajima’s D test (equal to zero at neutral equilibrium) (Tajima, 1989) was performed in MEGA version 11 (Tamura et al., 2021).

### Test for Recombination and Linkage Disequilibrium

Intragenic linkage disequilibrium (LD), and intragenic recombination rates were calculated by using DNA Sequence Polymorphism DnaSP version 6.12.03 (Rozas et al., 2017). Evidence of recombination was shown using the 4-gamete test to infer the minimum number of recombination events (Rm).

### Associations of Clinical Variables With Sequence Type or Clusters

Patients’ demographic and clinical data were recorded. Associations between genotypes and clinical variables were explored. The variables examined included age, sex, and geographical origin, source of isolates, infection status and predisposing factors (**Supplementary Table S2**). We investigated potential associations between sequence type and the variables using Pearson Chi Square test. Statistical analysis was performed using PASW Statistics 18 (SPSS Inc., Chicago, IL, USA) and STATA 15 (StataCorp LP, College Station, USA). *P-*values of <0.05 were considered statistically significant.

### Virulence Study

Virulence studies based upon *in vivo* survival in a murine model (Harun et al., 2010b) were performed. Eighteen strains listed in **Supplementary Table S2** (indicated by underlined strain numbers) and in **Figure 4** were selected to represent a wide global spectrum. Five seven-week-old female Balb/C mice were used, in which 0.2 ml of a conidial suspension (10^6^ conidia/ml) was inoculated intravenously *via* the lateral tail vein. The mice were monitored daily till 30 days post-inoculation for signs of infection, including ruffling of fur, inactivity, loss of weight, difficulty in breathing, and neurological signs such as ataxia. In accordance with the protocol approved by Western Sydney Local Health District Animal Ethics Committee (WSLHD AEC), mice that were deteriorating prior to that end point were sacrificed. Mean survival time (MST), estimated by Kaplan-Meier method, was used as a parameter to compare the relative pathogenicity among the selected strains. Comparison between each group was performed by the log-rank test as part of the software package PASW Statistics 18. Graphs were plotted using GraphPad Prism version 5.0b (GraphPad Software Inc., San Diego, USA). *P*-values of < 0.05 was considered as statistically significant.

## Results

### Selection of Genes for the *S. aurantiacum* MLST Scheme

DNA sequences of 24 genetic loci (*AAT, ACT, ANXC4, ATP6, BGT, BT2, EF1a, CAL, CAT, CHS, D1D2, FKS, LIP, GLN, IGS, MDH1, mtSSU, MP1, RPB1, RPB2, SOD2, TUB, VPS13*, and *ZRF*,) (**Supplementary Table S1**) were screened to assess their polymorphisms and hence suitability as candidate genetic loci for the S. aurantiacum MLST scheme. Following analysis of the DNA sequences obtained from 12 tester strains, the following six loci, actin (*ACT*), calmodulin (*CAL*), elongation factor 1-alpha (*EF1a*), RNA polymerase II subunit (*RPB2*), manganese superoxide dismutase (*SOD2*) and beta tubulin (*TUB*), were found to be the most variable, and were therefore selected to form the S. aurantiacum MLST scheme (http://mlst.mycologylab.org) (**Table 1**), and to identify the allele and sequence types of S. aurantiacum strains.

### Sequence Variability

The sizes of the six MLST gene fragments obtained including all gaps were: 830 bp for the *ACT* locus, 689 bp for the *CAL* locus, 715 bp for the *EF1α* locus, 952 bp for the *RPB2* locus, 437 bp for the *SOD2* locus and 401 bp for the *TUB* locus (**Table 2**). Seventy-seven (1.89%) polymorphic sites were identified across all six genes combined, which represents a total of 4,024 bp. The number of variable nucleotide sites per locus ranged between 5 (0.73%, *CAL*) and 18 (4.12%, *SOD2*). The variability among loci is shown in **Table 2**.

**Table 2:** Neutrality and genetic variability tests performed on each MLST locus.

Locus	No. of alleles	Length (bp)	Total Number of Sites^1^	No. of polymorphic sites (SNP)	No. of Haplotypes	*dN-dS*	*d_N_/d_S_^2^*	Nucleotide Diversity (π)	Haplotype Diversity (*Hd*)	Average no. of nucleotide differences (k)	Tajima’s D^3^	Tajima’s D (P-value)
*ACT*	22	830	824	12	12	-0.98	<1	0.00367	0.853	3.02401	0.87646	>0.10
*CAL*	8	689	689	5	8	-0.39	<1	0.00196	0.702	1.35305	0.63566	>0.10
*EF1a*	23	715	681	13	12	-0.12	<1	0.00277	0.695	1.88838	-0.54682	>0.10
*RPB2*	15	952	952	18	15	-3.62	<1	0.00518	0.864	4.93145	1.56775	>0.10
*SOD2*	18	437	433	18	16	0.81	>1	0.01335	0.770	5.78177	2.29468	<0.05
*TUB*	12	401	393	11	11	-0.93	<1	0.00517	0.582	2.03089	0.17512	>0.10
*Concatenated*	159 (ST)	4024	3972	77	149			0.00471	0.9965	18.67192	1.31347	>0.10

^1^Excluding sites with gaps/missing data.

^2^Non synonymous-synonymous substitutions ratio determined as described by Nei and Gojobori (1986) in MEGA version 11 (Tamura et al., 2021).

^3^Tajima’s test for neutrality (Tajima, 1989).

### Test for Selective Pressure, Variability, and Neutrality

The ratio of non-synonymous to synonymous nucleotide substitutions (*d_N_/d_S_*) was < 1 for five of the six MLST loci studied (**Table 2**). In all loci, the probability (p value was > 0.05 and therefore the null hypothesis of strict neutrality (*dN*=*dS*) was not rejected. All but one locus showed an *dN*-*dS* of negative value (*dN<dS*, *dN/dS* ratio of <1), thus indicating that with exception to *SOD2* none of the studied loci were under positive selective pressure (**Table 2**). The nucleotide diversity (π) ranged from 0.00196 for *CAL* to 0.01335 for *SOD2*, the haplotype diversity (*Hd*) ranged from 0.582 for *TUB* to 0.864 for *RPB2*, and the average number of nucleotide differences (k) ranged from 1.35305 for *CAL* to 5.78177 for *SOD2*, indicating an equal range for all loci, except for *SOD2* (**Table 2**). In the neutrality test, none of the Tajima’s D values obtained for the studied loci deviated significantly from zero, except for *SOD2*, suggesting the occurrence of balancing selection (Tajima, 1989) (**Table 2**).

### Recombination and Linkage Disequilibrium

The intragenic recombination test identified 1-4 recombination events (Rm) at the *ACT, EF1a, RPB2, SOD2*, and *TUB* loci, but no recombination at the *CAL* locus (**Table 3**). Based on the concatenated multilocus sequence data, the interlocus LD was assessed over all segregating sites using pairwise comparisons. The LD (|D′| Y = 0.8257–0.0970X) was detected with a negative slope, indicating a decrease in linkage with increased nucleotide distance. Of the 2556 pairwise comparisons, 798 were significant by the Fisher exact test, and 229 were significant after Bonferroni correction (**Table 3**).

**Table 3:** Pairwise interlocus linkage disequilibrium and recombination analysis of concatenated multilocus sequences from 188 *Scedosporium aurantiacum* strains.

Population	No. segregating sites analysed	No. pairwise comparisons	No. of pairs of sites with four gametic types	No. of significant pairwise comparisons^†^	Z_n_s*	Linkage disequilibrium (LD) value|D’|	Estimate of R/gene	Minimum no. recombination events (Rm)
All**	74	2556	1181	798 (229)	0.0505	Y = 0.8257 - 0.0970X	59.4	17
*ACT*	12	55	3	35 (23)	0.1585	Y = 1.0310 - 0.2161X	16	1
*CAL*	5	6	0	2 (2)	0.0337	Y = 1.0000 - 0.0000X	117	0
*EF1a*	13	66	6	18 (10)	0.0772	Y = 0.9131 + 0.2146X	4.6	2
*RPB2*	18	153	19	99 (71)	0.1571	Y = 0.9738 - 0.0082X	22.3	3
*SOD2*	18	153	30	97 (67)	0.2231	Y = 1.0359 - 0.5977X	5.5	4
*TUB*	11	55	4	12 (9)	0.1318	Y = 1.0016 - 0.1617X	0.8	1

^†^By Fisher’s exact test (after Bonferroni correction).

*Z_n_s, interlocus genetic association; |D’|, linkage disequilibrium (LD) value, where Y is LD value and X is nucleotide distance in kilobases.

**Based on concatenated multilocus gene sequence of all loci.

### Alleles and Sequence Type Distributions

The sequence alignments showed polymorphisms in all six loci denoting the presence of different alleles (**Table 2**). Each specific sequence was considered a unique allele type, which was then assigned a unique allele type number. For example, 22 alleles were defined for the *ACT* locus, which were assigned as allele types AT1 to AT22, accordingly. The *CAL* locus showed 8 alleles, the *EF1α* locus 23 alleles, the *RPB2* locus 15 alleles, the *SOD2* locus 18 alleles and the *TUB* locus 12 alleles (**Table 2**). A total of 159 sequence types were obtained by combining the allele types of the six MLST loci studied (**Supplementary Table S2**). Most of the strains exhibited unique sequence types (**Supplementary Table S2** and **Figures 2**, **3**). Only a few strains shared the same sequence type, but most of them originated from the same patient or from closely linked soil samples. For example, strains IHEM 23081 and IHEM 23092, which both exhibited the sequence type ST140, were recovered from respiratory secretions of the same patient at a one-year interval; likewise, six strains shared the sequence type ST108, *i.e.* 110349103-01/1, 110349103-01/2 and 110349103-01/3, as well as 110349211-01/1, 110349103-01/2 and 110349103/3, but they were recovered from two soil samples collected at the same location on the banks of the Loire river, in France. On the contrary, strains IHEM 23080 and IHEM 23081, which were recovered from the same clinical sample, exhibited distinct sequence types (ST 139 and ST140, respectively).

### Distribution of Sequence Types According to Geographical Origin

**Figure 1:**
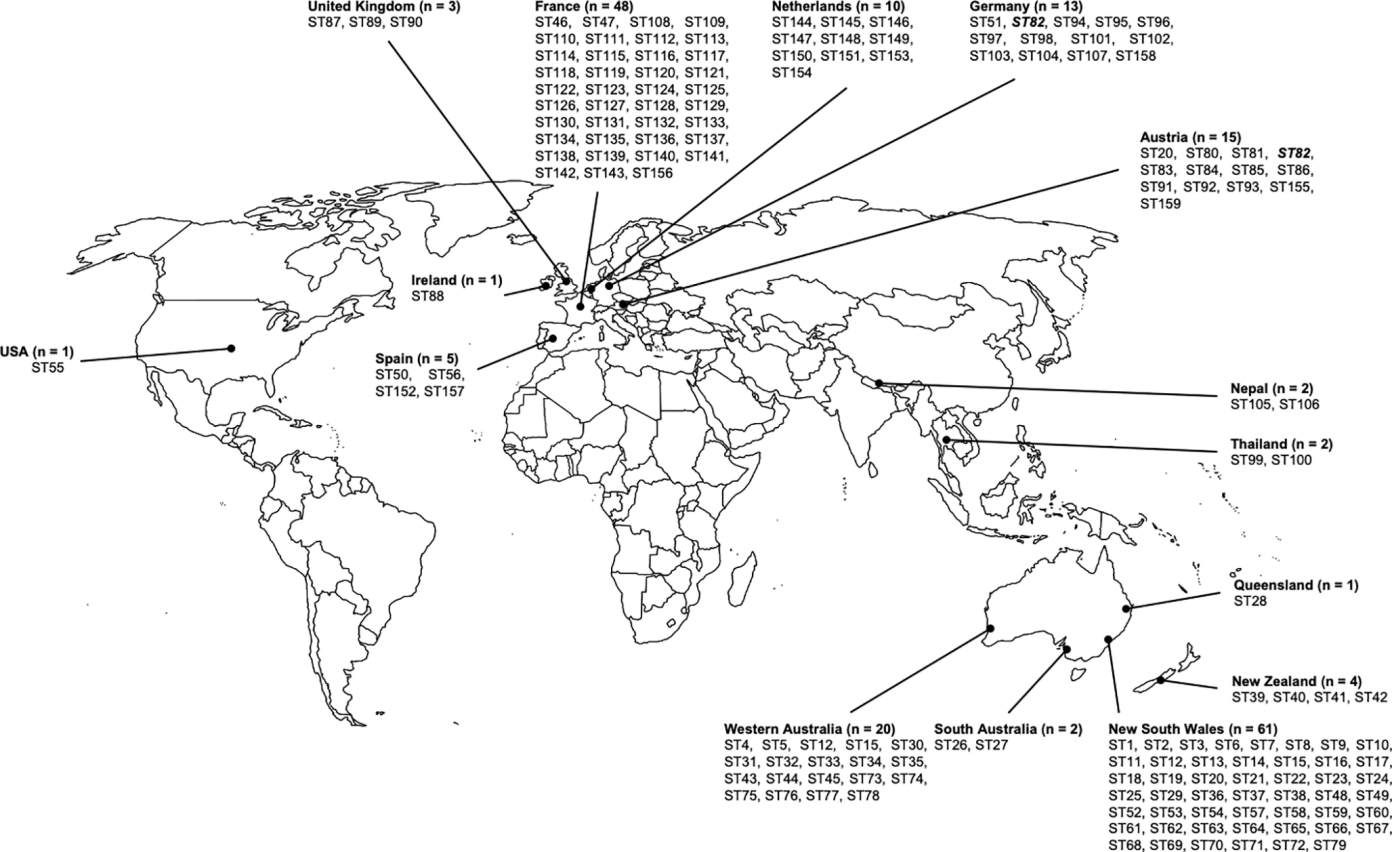
Geographic distribution of *Scedoporium aurantiacum* isolates. Distribution of studied *Scedosporium aurantiacum* isolates and their identified sequence types within Australia, New Zealand, Asia, Europe, and United States. ST's in bold and italics are the only ST shared between two countries.

**Figure 1** shows the distribution of sequence types according to the geographical origin. 69 of the 159 (43%) sequence types were only found in Australia, but none of them were predominant. Most of the Australian strains were obtained from New South Wales (NSW), suggesting a possible study bias. Among the 61 studied strains from NSW, 49 different sequence types were identified, most being specifically identified from this state since only two of these sequence types were also found outside NSW (ST12 and ST15, which were also found in Western Australia (WA)). Twenty strains collected in WA were studied, which revealed 19 sequence types, 17 of them being specifically identified from WA. ST26 and ST27 were found only in South Australia (SA), and ST28 was found only in Queensland (QLD) (**Figure 1** and **Supplementary Table S2**). The sequence types ST39, ST40, ST41 and ST42 were found exclusively in New Zealand.

Ninety-five strains collected in Europe were analyzed in this study. MLST analysis of these strains revealed a total number of 81 sequence types. Almost all of them were country specific, with the highest number of sequence types (39 STs) being found in France (**Figure 1** and **Supplementary Table S2**). A unique sequence type was identified from two distinct countries, *i.e.* the sequence type ST82, which was isolated from both Germany and Austria, with no obvious connection of the patients to each other.

Other sequence types obtained in this study include ST55, which was unique to the United States, ST99 and ST100, which were specific to Thailand, and ST105 and ST106, which were found in Nepal (**Figure 1** and **Supplementary Table S2**).

### Phylogenetic Relationships Among *S. aurantiacum* Strains

Parsimonious trees were constructed for each MLST locus (**Supplementary Figures S1**–**S6**). These analyses resulted in different tree topologies, indicating variable rates of gene evolution for each genetic locus (**Supplementary Figures S1** **–S6**).

**Figure 2:**
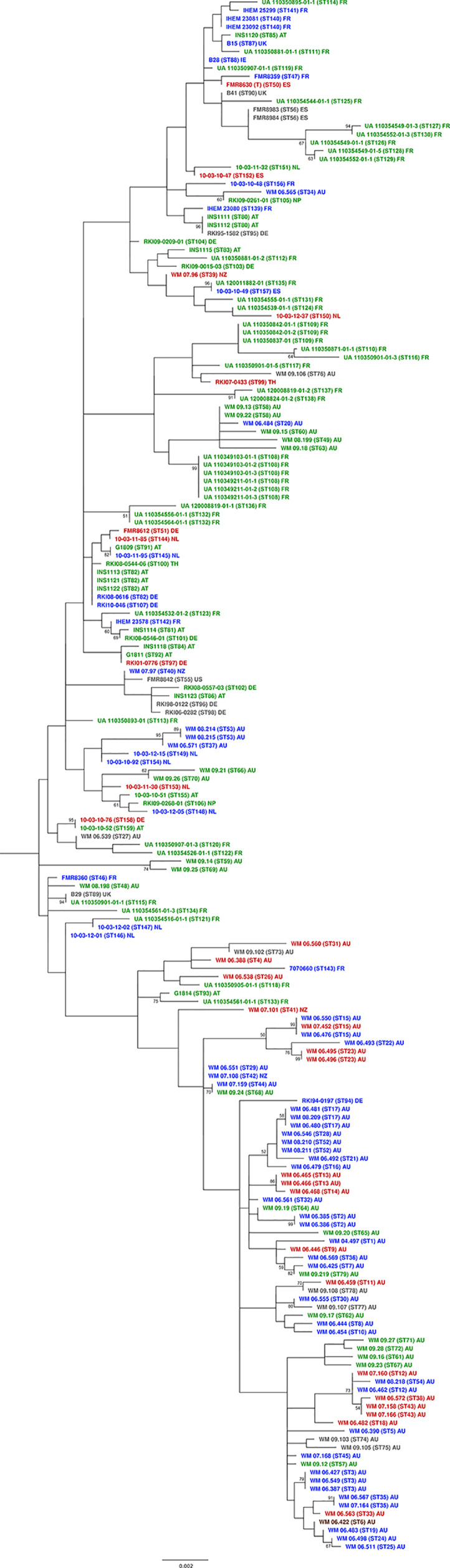
MLST maximum parsimony tree. Combined maximum parsimony tree for the six MLST loci (*ACT*, *CAL, EF1α*, *RPB2*, *SOD2* and *TUB*) generated from all obtained *Scedosporium aurantiacum* sequences using the program MEGA version 11. Environmental isolates (green), clinical invasive isolates (red), clinical colonizing isolates (blue), clinical isolates without information (grey), and veterinary isolates (brown). AT, Austria; AU, Australia; DE, Germany; ES, Spain; FR, France; UK, United Kingdom; IE, Ireland; NL, The Netherlands; NP, Nepal; NZ, New Zealand; TH, Thailand; and US, USA.

Maximum parsimony analysis of the combined gene sequences obtained from the six loci studied revealed a high genetic diversity amongst the 188 *S. aurantiacum* strains investigated (**Figure 2**), with most strains forming unique sequence types, which resulted in the widespread topology of the combined tree (**Figure 2** and **Supplementary Table S2**). Sequence types of the strains from different Australian states demonstrated no tendency to group with each other. When comparing the two major clusters (**Figure 2**), there was a significant difference according to the geographic origin of the strains (p < 0.0005). Most Australian strains belonged to cluster 2, while almost all European strains, except one German strain RKI94-0197, were grouped together in cluster 1 (**Figure 2**). The four isolates from New Zealand grouped either basal to cluster 1, which may be called ‘global cluster’ (WM 07.96, WM 07.97) or to cluster 2, the ‘Australian cluster’ (WM 07.101, WM 07.108) (**Figure 2**). In between the two major clusters the central basal group contains strains from Australia, Austria, France, Germany, Ireland, Netherlands, Spain, and the United Kingdom.

**Figure 3:**
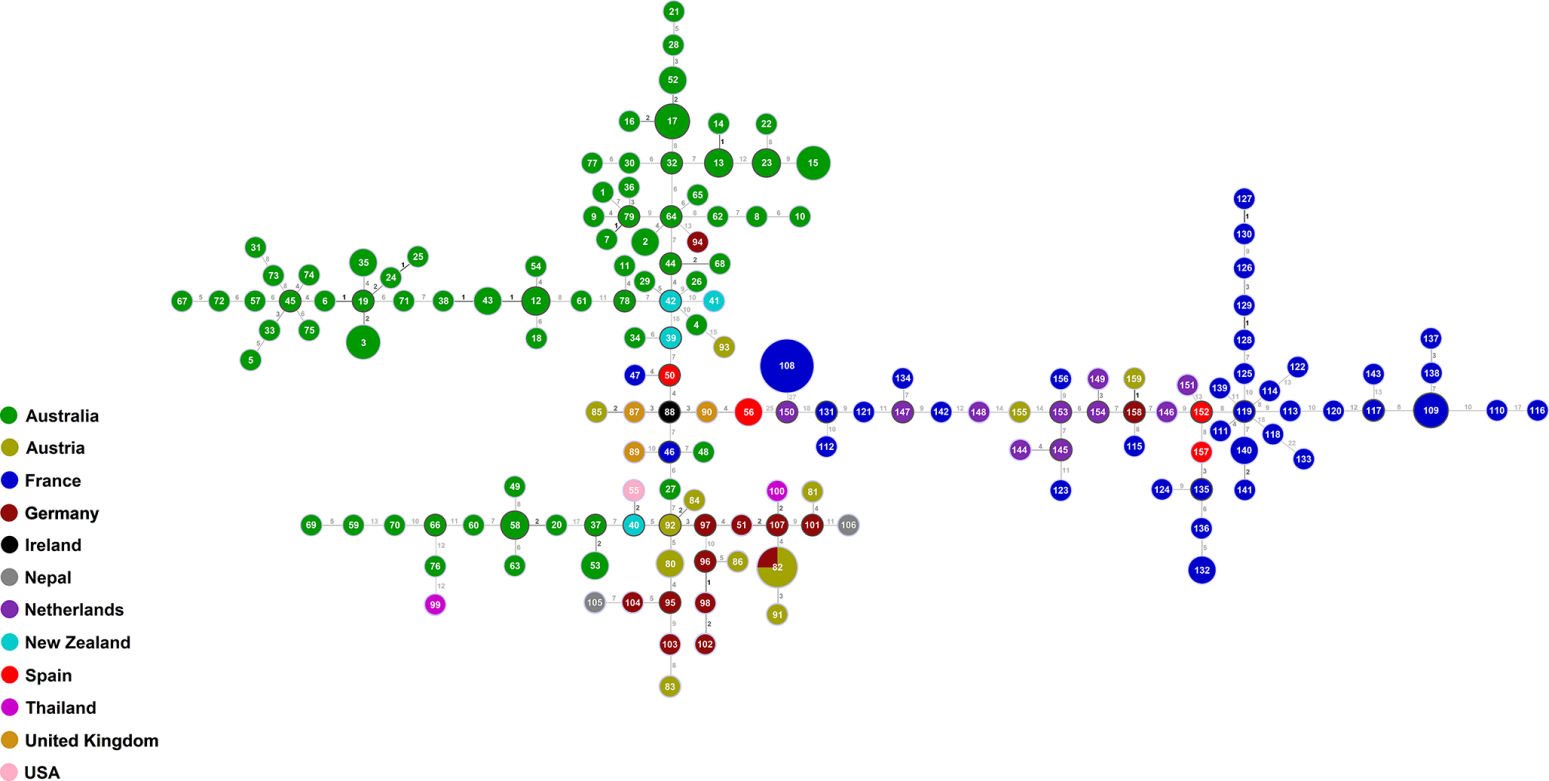
goeBURST minimum spanning tree for all 159 sequence types obtained from the combination of all allele types of the six MLST loci (*ACT*, *CAL, EF1α*, *RPB2*, *SOD2* and *TUB*) using the PHYLOViZ 2.0 analysis software, indicating the genetic relationships between all Australian and non-Australian *S. aurantiacum* isolates studied.

The goeBURST analysis of the obtained MLST data confirmed the high genetic diversity seen in the Australian *S. aurantiacum* population, as well as in the non-Australian population (**Figure 3**). Most of the Australian strains grouped separate to the European strains, forming a closely connected gene network. However, a subset of Australian strains was intermixed with and are closely related to the global strains, indicating certain genetic links between Australian and non-Australian strains (**Figure 3**). The results of the goeBURST analysis (**Figure 3**) confirmed the genetic relationships identified in the phylogenetic analysis of the combined genes as reflected in the concatenated gene tree (**Figures 2**, **3**).

Both clinical and environmental strains were widely distributed throughout the tree showing no sequence types to be indicative of a human clinical, veterinary, or environmental origin. Similar findings were found for the distribution of strains recovered from patients with either “invasive” disease or “colonization” (hereafter referred to as “invasive” or “colonizing” strains) (**Figure 2**).

### Association Between Genotype and Clinical Variables

Overall, the statistical analyses (PASW Statistics and STATA II) performed showed no significant associations between the different sequence types, which occurred largely as singletons, and clinical variables. In addition, there were no significant associations between a particular cluster and patient age (*p* = 0.078), sex (*p* = 0.076) or infection site (*p* > 0.05). Most analyzed strains were recovered from respiratory secretions from patients with chronic lung disease, such as cystic fibrosis; these strains that colonized the airways, were distributed throughout both clusters. There was only a single sequence type which was shared between colonizing and invasive strains, ST15, with WM 06.476 and WM 07.555 being colonizing strains, and WM 07.452 being an invasive strain.

When the two major clusters were compared with the patients predisposing factors (e.g., chronic lung disease, malignancy, diabetes, corticosteroid administration, chemotherapy, trauma, and drowning), there was no association between predisposing factors and any particular cluster (p > 0.05). An exception was a correlation of certain clusters and chronic lung disease, which was noted to show a significant difference (*p* = 0.005). In the “global cluster” there was a group of colonizing strains including WM 08.2114 and WM 08.215 (both ST53), WM 06.571 (ST37), 10-03-12 (ST149) and 10.03.10.92 (ST154), whereas the “Australian cluster” comprised another group of colonizing strains composed of WM 06.481, WM 08.209, and WM 06.480 (all ST17), WM 06.546, WM 08.210, and WM 08.211 (all ST52), WM 06.492 (ST21) and WM 06.479 (ST16) (**Figure 2**).

### Association Between Genotypes and Virulence in a Mouse Model

**Figure 4:**
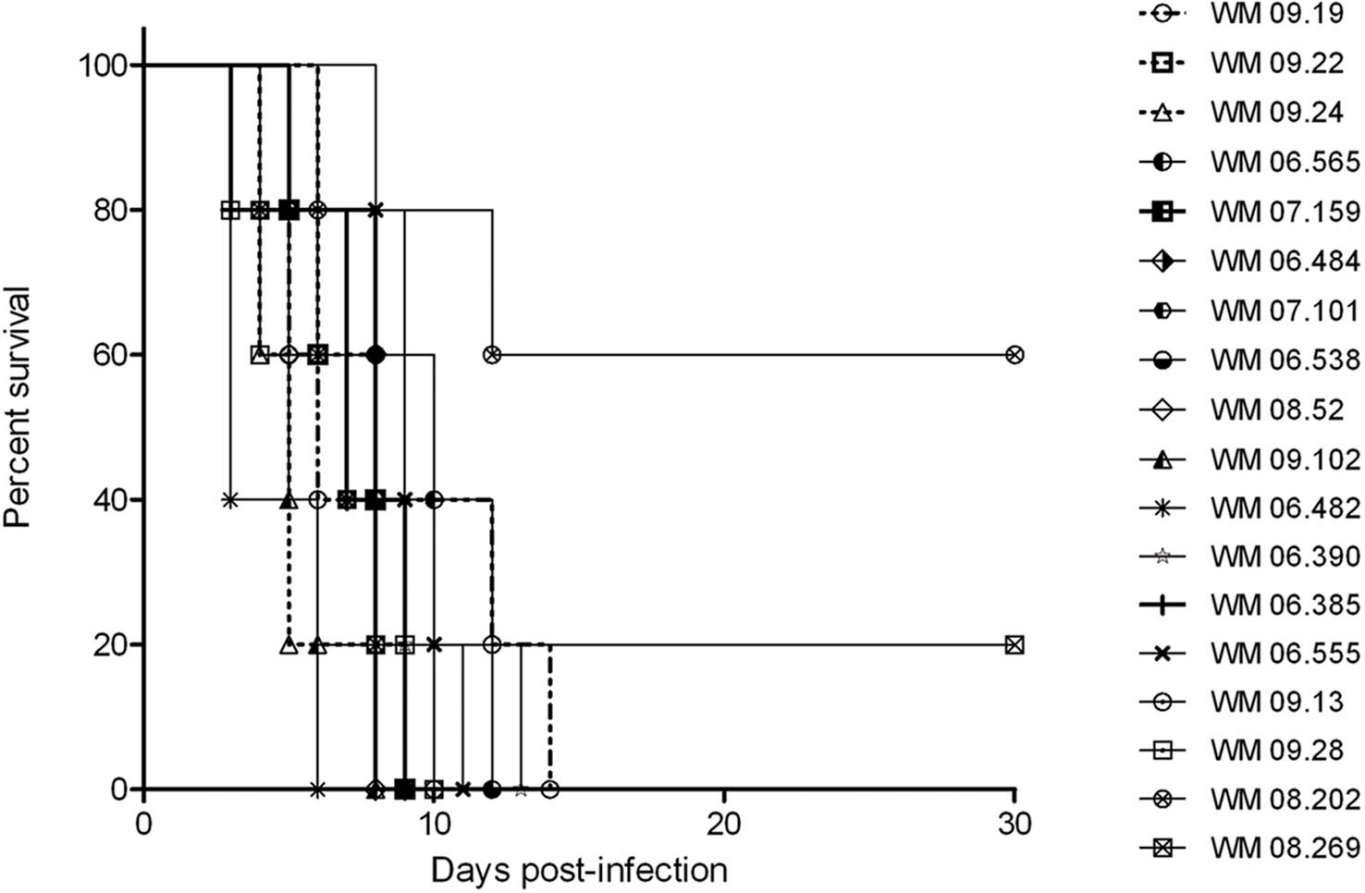
Animal virulence study. Survival plots of mice infected with selected *Scedosporium aurantiacum* strains. Environmental strains: WM 09.13 WM 09.19, WM 09.22, WM 09.24, and WM 09.28; Invasive clinical strains: WM 06.482, WM 06.538, WM 07.101, WM 08.202; Colonizing clinical strains: WM 06.385, WM 06.390, WM 06.484, WM 06.555, WM 06.565, WM 07.159, WM 08.52; and clinical strains without information on infection status: WM 08.269, WM 09.102.

The results of the survival analysis in mice, expressed as percent survival, for 18 *S. aurantiacum* strains are shown in **Figure 4**. All infected mice showed evidence of active infection (ruffling of fur, severe weight loss and neurological abnormalities, such as ataxia), as early as day 3 post-inoculation (**Figure 4**). An overall comparison between survival curves showed a significant difference (*p* = 0.007), with WM 06.482 being the most virulent strains followed by WM 09.24 and WM 08.52, and strains WM 08.269 and WM 08.202 being the least virulent strains. Of note, pairwise comparisons among all tested strains showed variable results. Pairwise comparison between invasive and colonizing strains, and between clinical and environmental strains revealed no significant difference.

## Discussion

MLST analysis, which allows for the accurate identification of discrete alleles for each analyzed locus, is a highly discriminatory tool for determining genetic variability between microbial strains (Bougnoux et al., 2002; Dodgson et al., 2003; Tavanti et al., 2005; Litvinseva et al., 2006; Bain et al., 2007; Jacobsen et al., 2007; Feng et al., 2008; Meyer et al., 2009; Debourgogne et al., 2010; Carriconde et al., 2011; Bernhard et al., 2013). The developed MLST scheme mirrors many MLST schemes for bacteria and fungi, which employed similar numbers of genetic loci (Maiden et al., 1998; Bougnoux et al., 2002; Dodgson et al., 2003; Tavanti et al., 2005; Litvinseva et al., 2006; Bain et al., 2007; Jacobsen et al., 2007; Feng et al., 2008; Odds and Jacobsen, 2008; Meyer et al., 2009; Debourgogne et al., 2010; Carriconde et al., 2011). In the present study, we applied a six-locus MLST approach to differentiate between *S. aurantiacum* strains with a high discrimination rate and for the first time to delineate the genetic variation amongst Australian *S. aurantiacum* strains in the context of a global strain population. Contrary to previous studies (Maiden et al., 1998; Odds and Jacobsen, 2008), the use of additional loci did not improve the resolution.

The herein newly developed MLST scheme, using six unrelated housekeeping genes (*ACT, CAL, EF1a, RPB2, SOD2* and *TUB*), was applied to investigate the population genetic structure of 188 environmental, clinical, and veterinary *S. aurantiacum* strains, mainly originating from Australia and Europe, together with a small number of North American, New Zealand and Asian strains. The MLST analysis revealed between 5-18 variable sites and 8-23 defined alleles per locus studied. Statistical analysis of the obtained dataset showed that the selected loci are suitable for a discriminatory *S. aurantiacum* MLST scheme (**Tables 2**, **3**). The ratio of non-synonymous to synonymous substitutions (*d_N_/d_S_*) was shown to be < 1 for five of the six loci, *i.e*. *ACT, CAL, EF1α*, *RPB2*, and *TUB*, indicating that these loci are not evolving under positive selection pressure (**Table 2**). Whilst a *d_N_/d_S_* value of > 1 was calculated for the *SOD2* locus, suggesting a positive selection pressure (**Table 2**), but the high number of polymorphic sites still warrants its inclusion in the new MLST scheme. The Tajima’s neutrality test demonstrated that five of the six genetic loci are not undergoing positive selection, except *SOD2*, suggesting that it is maybe going through balancing selection. The purpose of this test is to distinguish between a DNA sequence evolving randomly (“neutrally”) and a sequence evolving under non-random processes, including directional selection, or balancing selection, demographic expansion or contraction, genetic hitchhiking, or introgression. Randomly evolving DNA sequences contain mutations with no effect on the fitness and survival of an organism. The randomly evolving mutations are called “neutral”, while mutations under selection are “non-neutral” (Perez-Losada et al., 2006). Indeed, the obtained statistical result is an important finding since genes that are under selective pressure or diversifying selection, may exhibit highly polymorphic nucleotides and hence genetic variability that may possibly result in a false inference of the population structure of the organism studied. However, the nucleotide diversity (π) and the haplotype diversity (*Hd*) obtained for *SOD2*, as well as the obtained number of haplotypes, do still lay within the range of the other loci, warranting the inclusion of this locus in the new MLST scheme. The statistical values obtained for the five other MLST loci and the combined analysis of the concatenated sequences of all six loci, confirmed that the polymorphism identified for each of the loci included in the *S. aurantiacum* MLST scheme, resulting in a very high genetic diversity among the investigated strains, is not attributable to inappropriately selected MLST loci. The minimum number of recombination events and LD decay support evidence of genetic recombination, rather than clonal reproduction amongst the investigated strains. This is further supported by the nucleotide diversity (π), the relative high haplotype diversity (*Hd*), and the average number of nucleotide differences (k), showing that all loci contribute to the detection of the large number of polymorphic sites, 77 amongst the concatenated sequences of all six loci, manifested in the high genetic diversity of the studied *S. aurantiacum* population (**Table 2**). This is further supported by the derivation of 149 unique sequence types amongst 159 of the 188 strains. A similar method has been applied to evaluate recombination and linkage disequilibrium in other multilocus studies (Brown et al., 2004; Uraiwan et al., 2007).

In general, individual sequence analysis of each locus revealed a relatively low number of different allele types, and individual analysis of the selected loci resulted in contradicting gene topologies (**Supplementary Figures S1** **–S6**). This is not uncommon, as each of the genes evolves at different evolutionary rates (Rokas et al., 2003a; Rokas et al., 2003b; Planet, 2005). Datasets composed of multiple genes may have different histories and incongruence can be explained by genuine differences in the evolutionary process (Planet, 2005). As observed in the current study, the genetic variability may differ from one locus to another, with some loci demonstrating a higher polymorphism than others. Data presented show clearly, that the allele types for most of the genetic loci differ according to their geographic origin. Most allele types seen in Europe were rarely seen in Australia (**Supplementary Table S2**).

The combination of all six MLST loci resulted in a high discriminatory power, and consequently nearly all isolates investigated showed an individual sequence type. The phylogenetic analysis of the obtained sequences showed a very high genetic diversity in the Australian and non-Australian *S. aurantiacum* populations (**Figures 2**, **3**), with a total of 159 sequence types being derived from 188 strains studied (**Supplementary Table S2**). Further, strains originating from the same country likewise demonstrated substantial genetic variability (**Figures 1** **–3** and **Supplementary Table S2**). Possible explanations for this observation are, that the *S. aurantiacum* population is still undergoing active recombination as indicated by the incongruent topologies of the obtained individual gene trees (**Supplementary Figures S1** **–S6**), along with recombination tests and linkage disequilibrium analysis. Similar evidence has been shown in other genotyping studies, e.g., in *C. glabrata* (Dodgson et al., 2005; Lott et al., 2010), *C. neoformans* var. *grubii* (Litvinseva et al., 2006) and *C. gattii* (Carriconde et al., 2011), in which recombination and/or clonal expansion were demonstrated.

The clustering of the Australian versus the European strains/sequence types obtained from the available strains, the higher genetic diversity among the 84 Australian strains compared to the 95 European strains (**Table 4**), and the mix of some of the Australian strains within the “global cluster” suggest that the species *S. aurantiacum* maybe originated within the Australian continent and was subsequently dispersed to other parts of the world, as also indicated by the close genetic relationships between some of the Australian sequence types and those from other parts of the world revealed in the goeBURST analysis, while non-Australian sequence types are not interspaced within the main Australian sequence types clusters, except for the single German sequence type 94 (strain RKI95-0197) (**Figure 3**). However, to definitely identify the origin of this species, additional studies further expanding the number of *S. aurantiacum* strains from Africa, America and Asia are warranted.

**Table 4:** Comparison of neutrality and genetic variability of concatenated MLST sequences from Australia and Europe.

Geographic origin	No. of strains	No. of sequence types (ST)	Length (bp)	Total number of sites^1^	No. of polymorphic sites (SNP)	No. of haplotypes	Nucleotide diversity (π)	Haplotype diversity (*Hd*)	Average no. of nucleotide differences (k)	Tajima’s D^2^	Tajima’s D (P-value)
*Australia*	84	69	3994	3971	59	65	0.00381	0.993	15.12220	0.85368	>0.10
*Europe*	95	81	4022	3971	58	74	0.00365	0.991	14.47436	0.83132	>0.10

^1^Excluding sites with gaps/missing data

^2^Tajima’s test for neutrality (Tajima, 1989).

A key finding of this study is that clinical isolates were not genetically separated from the environmental isolates, whereby clinical isolates were present in all branches of the two major clusters, with some branches containing both clinical and environmental isolates. This infers that isolates from both sources are closely related, indicating that the environment may be the most likely source of colonization and subsequent infection. *Scedosporium* species have been reported globally (Rougeron et al., 2018), with *S. aurantiacum* being mainly reported from the environment in Australia (Harun et al., 2010a), Austria (Kaltseis et al., 2009), France (Rougeron et al., 2015), Morocco (Mouhajir et al., 2020) and Thailand (Luplertlop et al., 2016). However, the current analysis did not reveal any sequence type shared by environmental and clinical strains. Hence, it cannot be postulated that either infection or colonization is directly associated with a certain genotype of *S. aurantiacum* present in the environment.

Similarly, the current study did not show any clustering of either colonizing or invasive strains. However, some association was noted with some branches in the two main clusters harboring either mainly invasive strains or containing mainly colonizing strains. There were no genotypes which were identical or closely related between colonizing and invasive strains, except ST15 (colonizing strains WM 06.476, WM 07.555, and invasive strain WM 07.452). However, they are not directly related, as they have been isolated in 2004, 2005 and 2007, respectively (**Figure 2** and **Supplementary Table S2**). Both groups of strains can co-exist and have an equal possibility of colonizing the human host and subsequently causing invasive infection. Similar findings were made when the ability to degrade key elements of the complement cascade in the cerebrospinal fluid was investigated in correlation with the phylogenetic background, finding no phylogenetic grouping with the ability of a strain to degrade either the complement factors C3 or C1 (Rainer et al., 2011). Further studies are needed, on a wider range of clinical isolates systematically collected as part of a longitudinal clinical study to characterize the relatedness of colonizing and invasive isolates during progression to disease.

The lack of association between MLST genotype and infection sites in this study has also been noted in MLST studies of bacterial pathogens, such as *Streptococcus agalactiae* (Van der Mee-Marquet et al., 2008) and *Acinetobacter baumannii* (Sahl et al., 2011). In *C. albicans*, multi-locus sequence types were not associated with mating type, anatomical origin, or antifungal resistance (Chen et al., 2006). Apart from chronic lung disease, no significant association was seen for the other predisposing factors within specific branches of the two major clusters or with individual sequence types. However, interpretation of this study might be affected by missing data that resulted in a small sample analysis for both PASW Statistics and STATA II.

This study also attempted to find an association between different genotypes and virulence using a murine model. The major difference between the survival curves was obtained for the two clinical strains WM 08.202 and WM 08.269, for which end point survival rates were 20% and 40%, respectively. The other strains tested caused 100% mortality, with the clinical strain WM 06.482 inducing the highest mortality rate, followed by the environmental strain WM 09.24 and clinical strain WM 08.52, showing that highly virulent strains can circulate in the environment representing a potential risk of infections to humans. The lack of differences among the remaining strains suggests that most tested genotypes, regardless of their origin and clinical status, have a comparable degree of pathogenicity, and that genotype is not indicative of the virulence of a fungal strain as it has been shown for the molecular type VGII of the human pathogenic fungus *Cryptococcus gattii* (Ngamskulrungroj et al., 2011).

## Conclusions

The MLST typing scheme for *S. aurantiacum* developed as part of this study, is the first of its kind for *S. aurantiacum*. When applied to Australian and non-Australian strains it showed that this species is highly polymorphic. This MLST scheme offers a robust, reliable, and highly discriminatory molecular typing tool for *S. aurantiacum*. Together with the established database at http://mlst.mycology.org, it will enable data sharing and foster even greater international collaboration to enable an improved understanding of the *S. aurantiacum* population structure and to define the ultimate origin of the species. This will form the basis for further studies investigating the associations between genotypes and virulence or antifungal resistance, to facilitate more effective and tailored prevention and management strategies for patients at risk for infections by this emerging pathogen.

## Authors Contributions

WM, AH, J-PB, and SC conceived and designed the study. AH, AK, KS, FG, CF, and HL performed the experiments and data analysis. AH, HP, HL, SG, JK, MF, WB, ML, CB, IA, JR, JL, JA, KT, MS, CH, J-PB, SC, and WM collected, contributed strains and metadata to this study. AH, CH, J-PB, SC, and WM wrote the manuscript, with contributions and comments from all authors. All authors contributed to the article and approved the submitted version.

## Data Availability Statement

The datasets presented in this study can be found in online repositories. The names of the repository/repositories and accession number(s) can be found in the article/**Supplementary Material**.

## Ethics Statement

The animal study was reviewed and approved by Western Sydney Local Health District Animal Ethics Committee (#4194.06.012).

## Funding

The work was funded by an NHMRC project grant (APP1031943) to WM.

## Conflict of Interest

The authors declare that the research was conducted in the absence of any commercial or financial relationships that could be construed as a potential conflict of interest.

## Publisher’s Note

All claims expressed in this article are solely those of the authors and do not necessarily represent those of their affiliated organizations, or those of the publisher, the editors and the reviewers. Any product that may be evaluated in this article, or claim that may be made by its manufacturer, is not guaranteed or endorsed by the publisher.

## Supplementary Material

Supplementary Figure 1
*ACT* locus tree. Most parsimonious tree for the *ACT* locus for the 188 investigated *Scedosporium aurantiacum* isolates obtained with the program MEGA version 11 (numbers on the branches indicate bootstraps values above 50).

Supplementary Figure 2
*CAL* locus tree. Most parsimonious tree for the *CAL* locus for the 188 investigated *Scedosporium aurantiacum* isolates obtained with the program MEGA version 11 (numbers on the branches indicate bootstraps values above 50).

Supplementary Figure 3
*EF1α* locus tree. Most parsimonious tree for the *EF1α* locus for the 188 investigated *Scedosporium aurantiacum* isolates obtained with the program MEGA version 11 (numbers on the branches indicate bootstraps values above 50).

Supplementary Figure 4
*RPB2* locus tree. Most parsimonious tree for the *RPB2* locus for the 188 investigated *Scedosporium aurantiacum* isolates obtained with the program MEGA version 11 (numbers on the branches indicate bootstraps values above 50).

Supplementary Figure 5
*SOD2* locus tree. Most parsimonious tree for the *SOD2* locus for the 188 investigated *Scedosporium aurantiacum* isolates obtained with the program MEGA version 11 (numbers on the branches indicate bootstraps values above 50).

Supplementary Figure 6
*TUB* locus tree. Most parsimonious tree for the *TUB* locus for the 188 investigated *Scedosporium aurantiacum* isolates obtained with the program MEGA version 11 (numbers on the branches indicate bootstraps values above 50).

Supplementary Table 1 List of primers used in the MLST scheme development.

Supplementary Table 2 List of the studied *Scedosporium aurantiacum* strains, including strain number, strain information, origin, source of isolation, clinical data, MLST allele types (AT) of the six loci studied, sequence type (ST), and supplier.

Supplementary Table 3 GenBank accession numbers for all six genetic loci included in the *S. aurantiacum* MLST scheme for all investigated strains.
