# Supplementary figures and images for: Multilocus Sequence Typing Reveals Extensive Genetic Diversity of the Emerging Fungal Pathogen Scedosporium aurantiacum
Source: Front Cell Infect Microbiol. 2021 Dec 27;11:761596. doi: 10.3389/fcimb.2021.761596 (PMC8744116; doi:10.3389/fcimb.2021.761596)

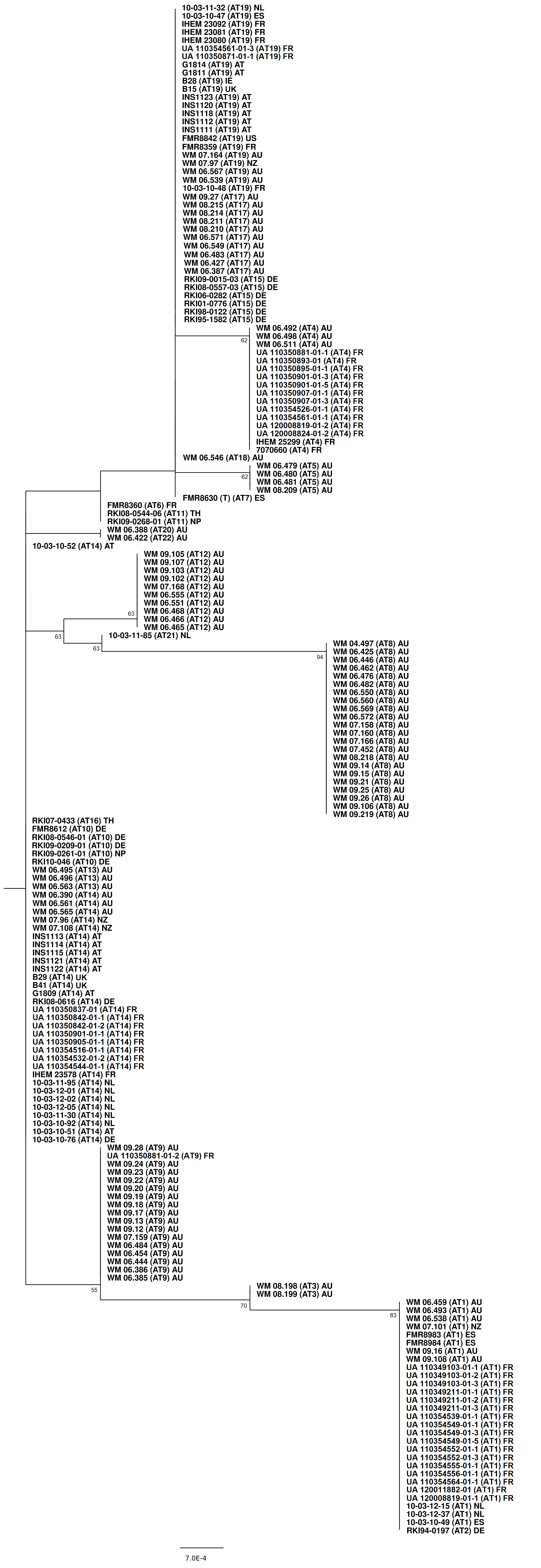

Supplement: Supplementary Figure 1 — ACT locus tree. Most parsimonious tree for the ACT locus for the 188 investigated Scedosporium aurantiacum isolates obtained with the program MEGA version 11 (numbers on the branches indicate bootstraps values above 50). [file Image_1.tif]

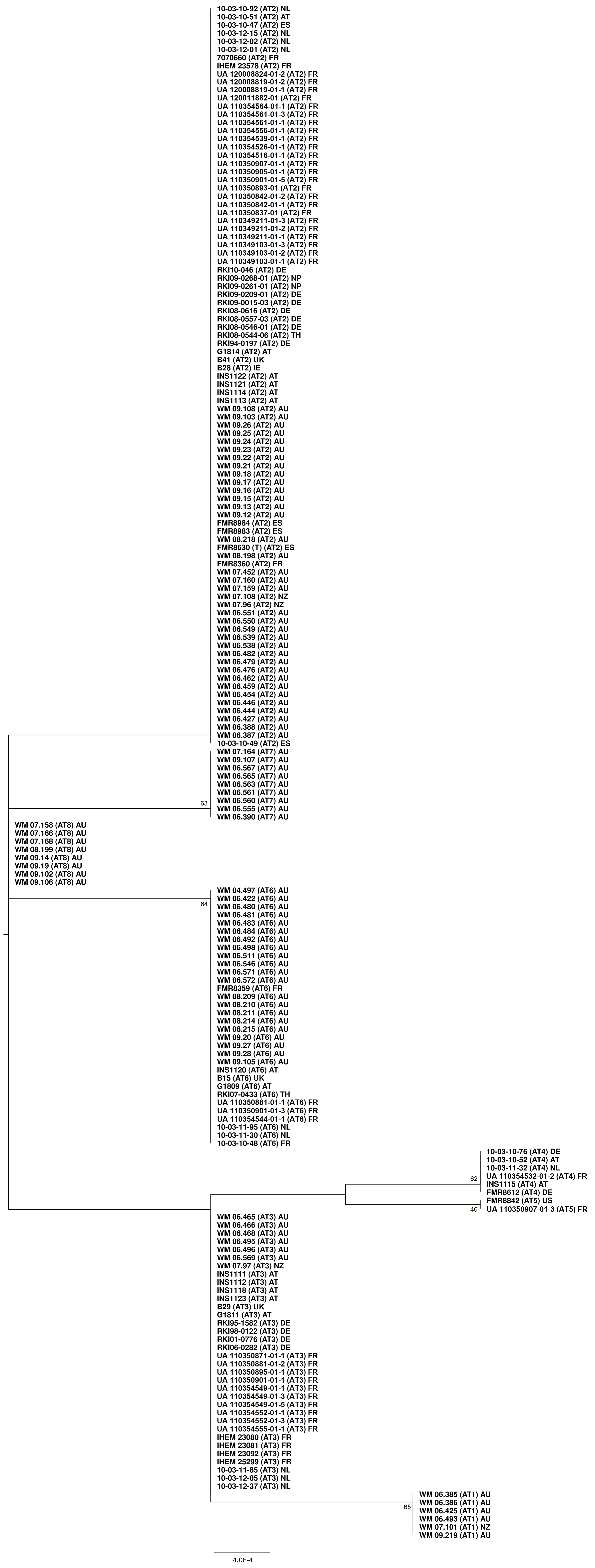

Supplement: Supplementary Figure 2 — CAL locus tree. Most parsimonious tree for the CAL locus for the 188 investigated Scedosporium aurantiacum isolates obtained with the program MEGA version 11 (numbers on the branches indicate bootstraps values above 50). [file Image_2.tif]

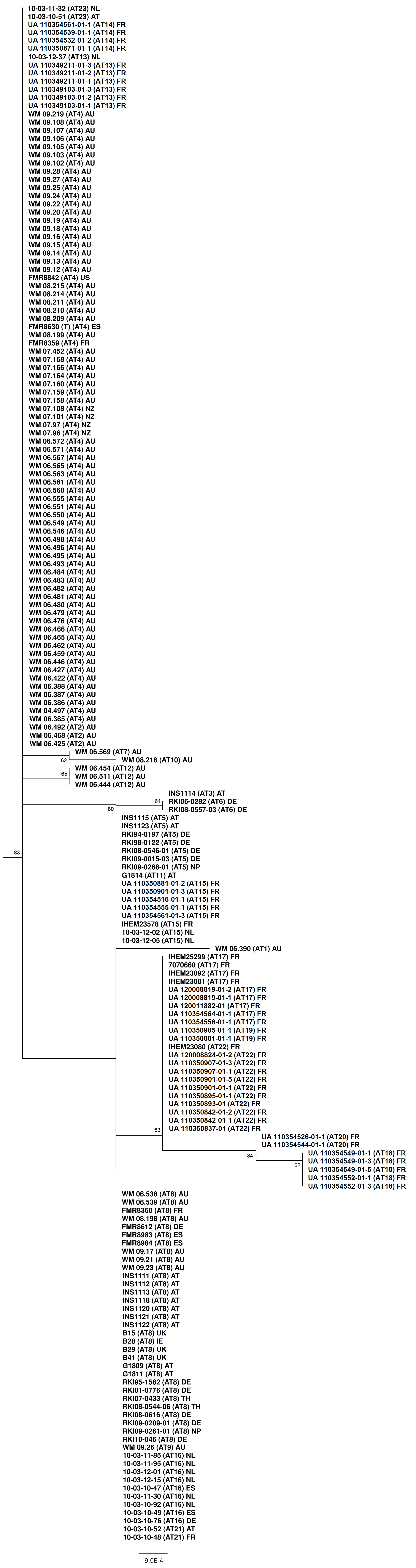

Supplement: Supplementary Figure 3 — EF1α locus tree. Most parsimonious tree for the EF1α locus for the 188 investigated Scedosporium aurantiacum isolates obtained with the program MEGA version 11 (numbers on the branches indicate bootstraps values above 50). [file Image_3.tif]

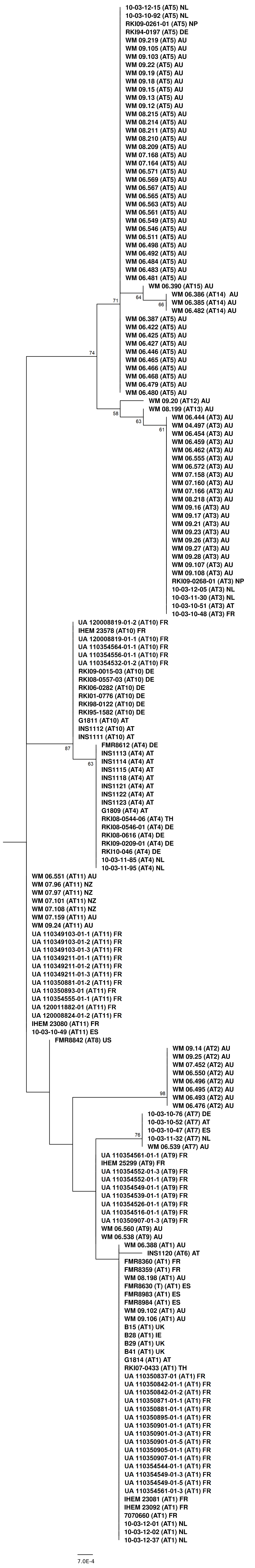

Supplement: Supplementary Figure 4 — RPB2 locus tree. Most parsimonious tree for the RPB2 locus for the 188 investigated Scedosporium aurantiacum isolates obtained with the program MEGA version 11 (numbers on the branches indicate bootstraps values above 50). [file Image_4.tif]

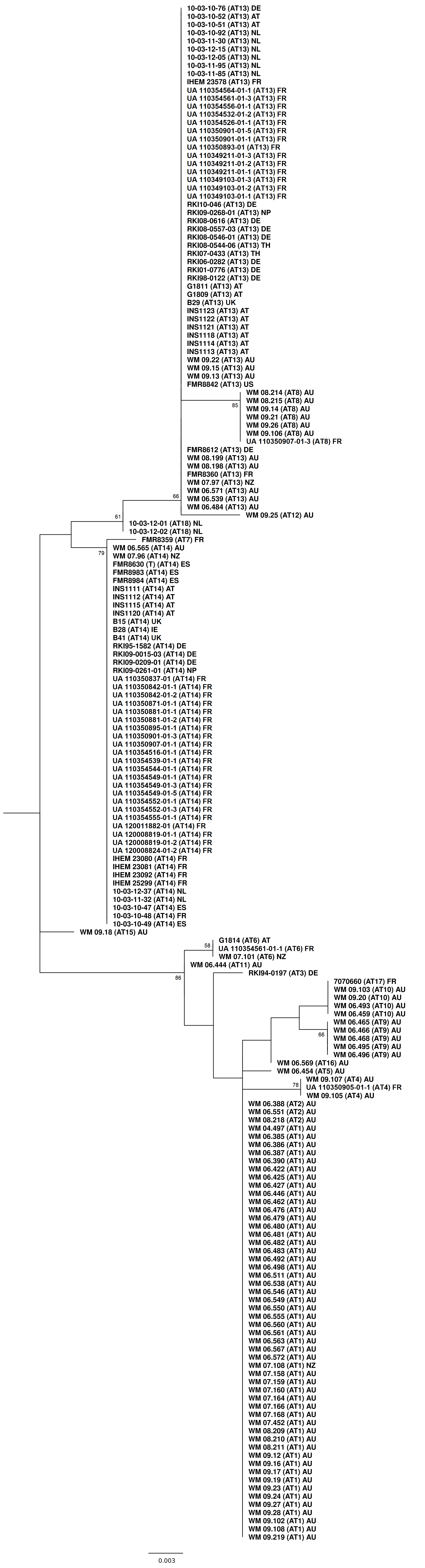

Supplement: Supplementary Figure 5 — SOD2 locus tree. Most parsimonious tree for the SOD2 locus for the 188 investigated Scedosporium aurantiacum isolates obtained with the program MEGA version 11 (numbers on the branches indicate bootstraps values above 50). [file Image_5.tif]

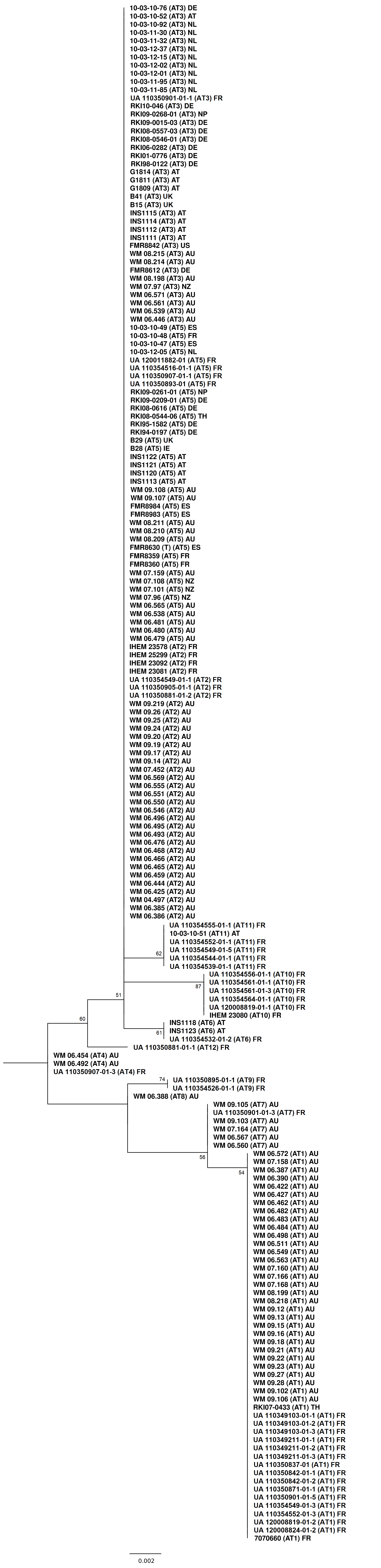

Supplement: Supplementary Figure 6 — TUB locus tree. Most parsimonious tree for the TUB locus for the 188 investigated Scedosporium aurantiacum isolates obtained with the program MEGA version 11 (numbers on the branches indicate bootstraps values above 50). [file Image_6.tif]
