# Supplementary material for: Multilocus Sequence Typing Reveals Extensive Genetic Diversity of the Emerging Fungal Pathogen Scedosporium aurantiacum
Source: Front Cell Infect Microbiol. 2021 Dec 27;11:761596. doi: 10.3389/fcimb.2021.761596 (PMC8744116; doi:10.3389/fcimb.2021.761596)
Supplement: Supplementary Table 1 — List of primers used in the MLST scheme development. [file Table_1.docx]

**Supplementary Table S1.** List of primers used in the MLST scheme development.

| **Locus name** | **Gene product** | **Primer sequences (from 5’ to 3’)*** | **Annealing temperature** | **Reference** |  |
| --- | --- | --- | --- | --- | --- |
| *ACT* | Actin | ACT-1: TGGGACGATATGGAIAAIATCTGGCA  ACT-4R: TCICGTATTCTTGCTTIGAIATCCACAT | 60 | Hoffman *et al*., 2007 | |
| *ATP6* | ATPase subunit 6 | ATP6-1: ATTAATTSWCCWTTAGAWCAATT  ATP6-2: TAATTCTANWGCATCTTTAATRTA | 60 | Kretzer and Bruns, 1999 | |
| *BT2* | Beta tubulin | BT2-F: ACCCTCRGTGTAGTGACCCTTGGC  BT2-R: ACCCTCRGTGTAGTGACCCTTGGC | 60 | Gilgado *et al.,* 2005 | |
| *EF1α* | Elongation factor -1 alpha | EF1: ATGGGTAAGGARGACAAGAC  EF2: GGARGTACCAGTSATCATGTT | 50 | O’Donnell *et al.*, 1998 | |
| *CAL* | Calmodulin | CL1A: TTTTTGCATCATGAGTTGGAC  CL2A: TTTTTGCATCATGAGTTGGAC | 52 | Gilgado *et al*., 2005 | |
| *FKS* | 1,3-beta glucan synthase | FKSF1: GTCAAATGCCACAACAACAACCT  FKSR1: AGCACTTCAGCAGCGTCTTCAG | 55 | Dodgson e*t al.*, 2003 | |
| *SOD2* | Manganese superoxide dismutase | SOD2F3: TCACCACGATAAACACCACC  SOD2R3: CGTCGATACCCAAGAGAGGA | 52 | Bernhard *et al.,* 2013 | |
| *CHS* | Chitin synthase | CHS-79F: TGGGGCAAGGATGCTTGGAAGAAG  CHS-354R: TGGAAGAACCATCTGTGAGAGTTG | 58 | Carbone and Kohn, 1999 | |
| *mtSSU* | Mitochondrial small subunit rDNA | MS3F: TAACGGCTGAACTGGCAAC  MS3R: CCTGCGTTGCAACATTACTC | 60 | O’Donnell *et al.,* 1998 | |
| *D1D2* | Large subunit rDNA | F63: GCATATCAATAAGCGGAGGAAAAG  LR3: GGTCCGTGTTTCAAGACGG | 50 | Scorzetti *et al.,* 2002 | |
| *TUB* | Beta tubulin | TUB2F: CTGTCCAACCCCTCTTACGGCGACCTGAAC  TUB2R: ACCCTCACCAGTATACCAATGCAAGAAAGC | 55 | Cruse *et al.,* 2002 | |
| *RPB1* | RNA polymerase I subunit | gRPB1-A: CNGCDATNTCRTTRTCCATRTA  fRPB1-C: NGCDATNTCRTTRTCCATRTA | 50-57 | Matheny *et al.,* 2002 | |
| *RPB2* | RNA polymerase II subunit | RPB2-5F: GAYGAYMGWGATCAYTTYGG  RPB2-7R: CCCATRGCTTGYTTRCCCAT | 55 | Liu *et al.,* 1999 | |
| *GLN* | Glutamine synthetase | GLN-F: GAGATAGTCAAGAATAAAAAAGT  GLN-R: ATCTCTTTCATCTTTTGGACC | 55 | Bougnoux *et al.,* 2002 | |
| *ZRF2* | Zinc transporter | ZRF2-F: CTCATCCAAGCTTGTTTCC  ZRF2-R: GTACCGCATCACCATCAA | 50 | Bain *et al.,* 2007 | |
| *VPS13* | Vacuolar protein sorting protein | VPS13-F: TCGTTGAGAGATATTCGACTT  VPS13-R: ACGGATGGATCTCCAGTCC | 55 | Bougnoux *et al.,* 2002 | |
| *AAT1* | Aspartate aminotransferase | AAT1-F: ACTCAAGCTAGATTTTTGGC  AAT1-R: CAGCAACATGATTAGCCC | 55 | Bougnoux *et a*l., 2002 | |
| *MP1* | Cell wall mannoprotein | MP1-F: CAAGCCCTCCAGAAAGGTATCCA  MP1-R: CTTTGTGGAGACCAATTCGCTGA | 50 | Lasker, 2006 | |
| *BGT1* | Beta-1,3-glucanosyl transferase | BGT1-F: GATCGGTTGCCAGTCTTTGA  BGT1-R: AATGGACGCAGAATGAAACT | 50 | Bain *et al.,* 2007 | |
| *LIP* | Lipase | LIP-F: CGCCTCACTTCTCCTCA  LIP-R: TGCGAAATGGCTGACG | 50 | Bain *et al.,* 2007 | |
| *CAT* | Catalase | CAT-F: AGCTCAACCGTCGTGAT  CAT-R: TGCCATGCCCAGACATA | 50 | Bain *et al.,* 2007 | |
| *IGS* | Intergenic spacer region | Ma-18S4- F: TAATGAGCCATTCGCAGTTTCGCTG  Ma-IGS1-R: CGTCACTTGTATTGGCAC | 52 | Pantou *et al.,* 2003 | |
| *ANXC4* | Annexin | ANXC4-F: GCGAGATAGCAACACTTCAGT  ANXC4-R: GGATACTGTTGCCCTAGATTTG | 50 | Bain *et al.,* 2007 | |

*S: C or G; W: A or T; N: A, C, G or T; R: A or G; Y: C or T; H: A, T or C; K: G or T; D: A, G or T.
