# Supplementary material for: Multilocus Sequence Typing Reveals Extensive Genetic Diversity of the Emerging Fungal Pathogen Scedosporium aurantiacum
Source: Front Cell Infect Microbiol. 2021 Dec 27;11:761596. doi: 10.3389/fcimb.2021.761596 (PMC8744116; doi:10.3389/fcimb.2021.761596)
Supplement: Supplementary Table 3 — GenBank accession numbers for all six genetic loci included in the S. aurantiacum MLST scheme for all investigated strains. [file Table_3.doc]

**Supplementary Table S3.** GenBank accession numbers for all six genetic loci included in the *S. aurantiacum* MLST scheme for all investigated strains.

| Strain no. | GenBank Accession no. | | | | | |
| --- | --- | --- | --- | --- | --- | --- |
|  | *ACT* | *CAL* | *EF1a* | *RPB2* | *SOD2* | *TUB* |
| WM04.497 | JX103855 | JX103831 | JX444716 | JQ754521 | JX120624 | JX267194 |
| WM06.385 | JX293567 | JX456058 | JX444715 | JQ754522 | JX120625 | JX267195 |
| WM06.386 | JX293568 | JX456059 | JX444931 | JQ754523 | JX120626 | JX267196 |
| WM06.387 | JX444704 | JX463755 | JX444932 | JQ754524 | JX120627 | JX463895 |
| WM06.388 | JX103856 | JX463756 | JX444933 | JQ754525 | JX267220 | JX463862 |
| WM06.390 | JX103857 | JX463979 | JX463947 | JQ754526 | JX120628 | JX463896 |
| WM06.422 | JX444705 | JX103832 | JX444934 | JQ754527 | JX120629 | JX463897 |
| WM06.425 | JX103858 | JX456060 | JX463966 | JQ754528 | JX120630 | JX267197 |
| WM06.427 | JX444706 | JX463757 | JX444935 | JQ754529 | JX120631 | JX463898 |
| WM06.444 | JX293569 | JX463758 | JX463948 | JQ754530 | JX464003 | JX267198 |
| WM06.446 | JX463859 | JX463759 | JX444936 | JQ754531 | JX120632 | JX463867 |
| WM06.454 | JX293570 | JX463760 | JX463949 | JQ754532 | JX464004 | JX472450 |
| WM06.459 | JX103859 | JX463761 | JX444937 | JQ754533 | JX464005 | JX267199 |
| WM06.462 | JX103860 | JX463762 | JX444938 | JQ754534 | JX120633 | JX463899 |
| WM06.465 | JX103861 | JX463811 | JX444939 | JQ754535 | JX464006 | JX267200 |
| WM06.466 | JX103862 | JX463812 | JX444940 | JQ754536 | JX464007 | JX267201 |
| WM06.468 | JX103863 | JX463813 | JX463967 | JQ754537 | JX464008 | JX267202 |
| WM06.476 | JX103864 | JX463763 | JX444941 | JQ754538 | JX120634 | JX267203 |
| WM06.479 | JX103865 | JX463764 | JX444942 | JQ754539 | JX120635 | JX463830 |
| WM06.480 | JX103866 | JX103833 | JX444943 | JQ754540 | JX120636 | JX463831 |
| WM06.481 | JX103867 | JX103834 | JX444944 | JQ754541 | JX120637 | JX463832 |
| WM06.482 | JX103868 | JX463765 | JX444945 | JQ754542 | JX120638 | JX463900 |
| WM06.483 | JX444707 | JX103835 | JX444946 | JQ754543 | JX120639 | JX463901 |
| WM06.484 | JX293571 | JX103836 | JX444947 | JQ754544 | JX293599 | JX463902 |
| WM06.492 | JX103869 | JX103837 | JX463968 | JQ754545 | JX120640 | JX463865 |
| WM06.493 | JX103870 | JX456061 | JX444948 | JQ754546 | JX267221 | JX267204 |
| WM06.495 | JX103871 | JX463814 | JX444949 | JQ754547 | JX464009 | JX267205 |
| WM06.496 | JX103872 | JX463815 | JX444950 | JQ754548 | JX464010 | JX267206 |
| WM06.498 | JX103873 | JX103838 | JX444951 | JQ754549 | JX120641 | JX463903 |
| WM06.511 | JX103874 | JX103839 | JX463950 | JQ754550 | JX120642 | JX463904 |
| WM06.538 | JX103875 | JX463766 | JX456043 | JQ754551 | JX120643 | JX463833 |
| WM06.539 | JX103876 | JX463767 | JX456044 | JQ754552 | JX293600 | JX463868 |
| WM06.546 | JX103877 | JX103840 | JX463951 | JQ754553 | JX120644 | JX463866 |
| WM06.549 | JX444708 | JX463768 | JX463952 | JQ754554 | JX120645 | JX463905 |
| WM06.550 | JX103878 | JX463769 | JX463953 | JQ754555 | JX120646 | JX267207 |
| WM06.551 | JX103879 | JX463770 | JX444953 | JQ754556 | JX267222 | JX267208 |
| WM06.555 | JX103880 | JX463980 | JX444954 | JQ754557 | JX120647 | JX267209 |
| WM06.560 | JX103881 | JX463981 | JX444955 | JQ754558 | JX120648 | JX463906 |
| WM06.561 | JX103882 | JX463982 | JX444956 | JQ754559 | JX120649 | JX463869 |
| WM06.563 | JX103883 | JX463983 | JX444957 | JQ754560 | JX120650 | JX463907 |
| WM06.565 | JX103884 | JX463984 | JX444958 | JQ754561 | JX293601 | JX463834 |
| WM06.567 | JX103885 | JX463985 | JX444959 | JQ754562 | JX120651 | JX463908 |
| WM06.569 | JX103886 | JX463816 | JX463954 | JQ754563 | JX267223 | JX267210 |
| WM06.571 | JX444709 | JX103841 | JX456009 | JQ754564 | JX464001 | JX463870 |
| WM06.572 | JX103887 | JX103842 | JX456010 | JQ754565 | JX120652 | JX463909 |
| WM07.96 | JX103888 | JX463771 | JX456011 | JQ754566 | JX293602 | JX463835 |
| WM07.97 | JX103889 | JX463817 | JX456012 | JQ754567 | JX293603 | JX463871 |
| WM07.101 | JX103890 | JX456062 | JX456013 | JQ754568 | JX464011 | JX463836 |
| WM07.108 | JX103891 | JX463772 | JX456014 | JQ754569 | JX120653 | JX463837 |
| WM07.158 | JX103892 | JX463986 | JX456015 | JQ754570 | JX120654 | JX463910 |
| WM07.159 | JX293572 | JX463773 | JX456016 | JQ754571 | JX464012 | JX463838 |
| WM07.160 | JX103893 | JX463774 | JX456017 | JQ754572 | JX120656 | JX463911 |
| WM07.164 | JX103894 | JX463987 | JX463955 | JQ754573 | JX120657 | JX463912 |
| WM07.166 | JX103895 | JX463988 | JX456018 | JQ754574 | JX120658 | JX463913 |
| WM07.168 | JX103896 | JX463989 | JX456019 | JQ754575 | JX120659 | JX463914 |
| WM07.452 | JX103897 | JX463775 | JX456020 | JQ754576 | JX120660 | JX267211 |
| WM08.51 | JX463860 | JX463776 | JX456045 | JQ754577 | JX293604 | JX463839 |
| WM08.52 | JX103898 | JX103843 | JX463956 | JQ754578 | JX293605 | JX463840 |
| WM08.198 | JX103900 | JX463777 | JX456046 | JQ754579 | JX293606 | JX463872 |
| WM08.199 | JX103899 | JX463990 | JX456021 | JQ754580 | JX293607 | JX463915 |
| WM08.202 | JX463861 | JX463778 | JX456022 | JQ754581 | JX464002 | JX463841 |
| WM08.203 | JX293573 | JX463991 | JX463932 | JQ754582 | JX293608 | JX463873 |
| WM08.209 | JX103901 | JX103844 | JX456023 | JQ754583 | JX120661 | JX463842 |
| WM08.210 | JX444710 | JX103845 | JX456026 | JQ754584 | JX120662 | JX463843 |
| WM08.211 | JX444711 | JX103846 | JX456027 | JQ754585 | JX120663 | JX463852 |
| WM08.214 | JX444712 | JX103847 | JX456024 | JQ754586 | JX293609 | JX463874 |
| WM08.215 | JX444713 | JX103848 | JX456025 | JQ754587 | JX293610 | JX463875 |
| WM08.218 | JX103902 | JX463779 | JX463957 | JQ754588 | JX267224 | JX463916 |
| WM08.269 | JX103903 | JX463992 | JX456028 | JQ754589 | JX293611 | JX463876 |
| WM08.270 | JX103904 | JX463780 | JX456047 | JQ754590 | JX293612 | JX463844 |
| WM08.271 | JX103905 | JX463781 | JX456048 | JQ754591 | JX293613 | JX463845 |
| WM09.12 | JX293574 | JX463782 | JX456029 | JQ754592 | JX120664 | JX463917 |
| WM09.13 | JX293575 | JX463783 | JX456030 | JQ754593 | JX293614 | JX463918 |
| WM09.14 | JX103906 | JX463993 | JX456031 | JQ754594 | JX293615 | JX267212 |
| WM09.15 | JX103907 | JX463784 | JX456032 | JQ754595 | JX293616 | JX463919 |
| WM09.16 | JX103908 | JX463785 | JX456033 | JQ754596 | JX120665 | JX463920 |
| WM09.17 | JX293576 | JX463786 | JX456049 | JQ754597 | JX120666 | JX267213 |
| WM09.18 | JX293577 | JX463787 | JX463958 | JQ754598 | JX293617 | JX463921 |
| WM09.19 | JX293578 | JX463994 | JX456034 | JQ754599 | JX120667 | JX267214 |
| WM09.20 | JX293579 | JX103849 | JX456035 | JQ754600 | JX267225 | JX267215 |
| WM09.21 | JX103909 | JX463788 | JX456050 | JQ754601 | JX293618 | JX463922 |
| WM09.22 | JX293580 | JX463789 | JX456036 | JQ754602 | JX293619 | JX463923 |
| WM09.23 | JX293581 | JX463790 | JX456051 | JQ754603 | JX120668 | JX463924 |
| WM09.24 | JX293582 | JX463791 | JX456037 | JQ754604 | JX120669 | JX267216 |
| WM09.25 | JX103910 | JX463792 | JX463959 | JQ754605 | JX293620 | JX267217 |
| WM09.26 | JX103911 | JX463793 | JX463960 | JQ754606 | JX293621 | JX267218 |
| WM09.27 | JX444714 | JX103850 | JX456038 | JQ754607 | JX120670 | JX463925 |
| WM09.28 | JX293583 | JX103851 | JX456039 | JQ754608 | JX120671 | JX463926 |
| WM09.102 | JX103912 | JX463995 | JX463961 | JQ754609 | JX120672 | JX463927 |
| WM09.103 | JX103913 | JX463996 | JX456040 | JQ754610 | JX267226 | JX463928 |
| WM09.105 | JX103914 | JX103852 | JX456041 | JQ754611 | JX267227 | JX463929 |
| WM09.106 | JX103915 | JX463997 | JX463962 | JQ754612 | JX293622 | JX463930 |
| WM09.107 | JX103916 | JX463998 | JX456042 | JQ754613 | JX267228 | JX463846 |
| WM09.108 | JX103917 | JX463999 | JX463963 | JQ754614 | JX120673 | JX463847 |
| WM09.219 | JX103918 | JX456063 | JX463964 | JQ754615 | JX120674 | JX267219 |
| WM10.127 | JX103919 | JX463818 | JX456052 | JQ754616 | JX293623 | JX463877 |
| WM10.128 | JX103920 | JX463819 | JX456053 | JQ754617 | JX293624 | JX463878 |
| WM10.129 | JX103921 | JX463794 | JX456054 | JQ754618 | JX293625 | JX463848 |
| WM10.130 | JX103922 | JX463795 | JX463969 | JQ754619 | JX293626 | JX463879 |
| WM10.131 | JX103923 | JX464000 | JX463970 | JQ754620 | JX293627 | JX463880 |
| WM10.134 | JX103924 | JX463820 | JX456055 | JQ754621 | JX293628 | JX463881 |
| WM10.136 | JX103925 | JX103853 | JX456056 | JQ754622 | JX293629 | JX463849 |
| WM10.137 | JX103926 | JX463796 | JX472449 | JQ754623 | JX293630 | JX463850 |
| WM10.138 | JX103927 | JX463797 | JX456057 | JQ754624 | JX293631 | JX463851 |
| WM10.139 | JX103928 | JX463821 | JX463971 | JQ754625 | JX293632 | JX463882 |
| WM10.140 | JX103929 | JX463822 | JX463933 | JQ754626 | JX293633 | JX463883 |
| WM10.141 | JX103930 | JX463798 | JX463934 | JQ754627 | JX293634 | JX463863 |
| WM10.142 | JX103931 | JX463823 | JX463935 | JQ754628 | JX293635 | JX463864 |
| WM10.143 | JX103932 | JX463799 | JX463936 | JQ754629 | JX293636 | JX472451 |
| WM10.144 | JX103933 | JX463824 | JX463937 | JQ754630 | JX293637 | JX463884 |
| WM10.145 | JX103934 | JX463825 | JX463938 | JQ754631 | JX293638 | JX463885 |
| WM10.146 | JX103935 | JX463800 | JX463965 | JQ754632 | JX464013 | JX463886 |
| WM11.43 | JX293584 | JX463801 | JX463972 | JQ754633 | JX464014 | JX463853 |
| WM11.44 | JX293585 | JX463826 | JX463939 | JQ754634 | JX293639 | JX463854 |
| WM11.45 | JX293586 | JX463827 | JX463973 | JQ754635 | JX293640 | JX463887 |
| WM11.46 | JX293587 | JX463828 | JX463940 | JQ754636 | JX293641 | JX463888 |
| WM11.47 | JX293588 | JX463829 | JX463974 | JQ754637 | JX293642 | JX463889 |
| WM11.48 | JX293589 | JX103854 | JX463941 | JQ754638 | JX293643 | JX463931 |
| WM11.50 | JX293590 | JX463802 | JX463942 | JQ754639 | JX293644 | JX463855 |
| WM11.51 | JX293591 | JX463803 | JX463975 | JQ754640 | JX293645 | JX463890 |
| WM11.52 | JX293592 | JX463804 | JX463976 | JQ754641 | JX293646 | JX463891 |
| WM11.53 | JX293593 | JX463805 | JX463943 | JQ754642 | JX293647 | JX463856 |
| WM11.54 | JX293594 | JX463806 | JX463977 | JQ754643 | JX293648 | JX463892 |
| WM11.55 | JX293595 | JX463807 | JX463944 | JQ754644 | JX293649 | JX463857 |
| WM11.56 | JX293596 | JX463808 | JX463945 | JQ754645 | JX293650 | JX463858 |
| WM11.57 | JX293597 | JX463809 | JX463978 | JQ754646 | JX293651 | JX463893 |
| WM11.59 | JX293598 | JX463810 | JX463946 | JQ754647 | JX293652 | JX463894 |
| WM 13.319 | KJ783933 | KJ783994 | KJ784055 | KJ784116 | KJ784177 | KJ784238 |
| WM 13.320 | KJ783934 | KJ783995 | KJ784056 | KJ784117 | KJ784178 | KJ784239 |
| WM 13.321 | KJ783935 | KJ783996 | KJ784057 | KJ784118 | KJ784179 | KJ784240 |
| WM 13.322 | KJ783936 | KJ783997 | KJ784058 | KJ784119 | KJ784180 | KJ784241 |
| WM 13.323 | KJ783937 | KJ783998 | KJ784059 | KJ784120 | KJ784181 | KJ784242 |
| WM 13.324 | KJ783938 | KJ783999 | KJ784060 | KJ784121 | KJ784182 | KJ784243 |
| WM 13.325 | KJ783939 | KJ784000 | KJ784061 | KJ784122 | KJ784183 | KJ784244 |
| WM 13.326 | KJ783940 | KJ784001 | KJ784062 | KJ784123 | KJ784184 | KJ784245 |
| WM 13.327 | KJ783941 | KJ784002 | KJ784063 | KJ784124 | KJ784185 | KJ784246 |
| WM 13.328 | KJ783942 | KJ784003 | KJ784064 | KJ784125 | KJ784186 | KJ784247 |
| WM 13.329 | KJ783943 | KJ784004 | KJ784065 | KJ784126 | KJ784187 | KJ784248 |
| WM 13.330 | KJ783944 | KJ784005 | KJ784066 | KJ784127 | KJ784188 | KJ784249 |
| WM 13.331 | KJ783945 | KJ784006 | KJ784067 | KJ784128 | KJ784189 | KJ784250 |
| WM 13.332 | KJ783946 | KJ784007 | KJ784068 | KJ784129 | KJ784190 | KJ784251 |
| WM 13.333 | KJ783947 | KJ784008 | KJ784069 | KJ784130 | KJ784191 | KJ784252 |
| WM 13.334 | KJ783948 | KJ784009 | KJ784070 | KJ784131 | KJ784192 | KJ784253 |
| WM 13.335 | KJ783949 | KJ784010 | KJ784071 | KJ784132 | KJ784193 | KJ784254 |
| WM 13.336 | KJ783950 | KJ784011 | KJ784072 | KJ784133 | KJ784194 | KJ784255 |
| WM 13.337 | KJ783951 | KJ784012 | KJ784073 | KJ784134 | KJ784195 | KJ784256 |
| WM 13.338 | KJ783952 | KJ784013 | KJ784074 | KJ784135 | KJ784196 | KJ784257 |
| WM 13.339 | KJ783953 | KJ784014 | KJ784075 | KJ784136 | KJ784197 | KJ784258 |
| WM 13.340 | KJ783954 | KJ784015 | KJ784076 | KJ784137 | KJ784198 | KJ784259 |
| WM 13.341 | KJ783955 | KJ784016 | KJ784077 | KJ784138 | KJ784199 | KJ784260 |
| WM 13.342 | KJ783956 | KJ784017 | KJ784078 | KJ784139 | KJ784200 | KJ784261 |
| WM 13.343 | KJ783957 | KJ784018 | KJ784079 | KJ784140 | KJ784201 | KJ784262 |
| WM 13.344 | KJ783958 | KJ784019 | KJ784080 | KJ784141 | KJ784202 | KJ784263 |
| WM 13.345 | KJ783959 | KJ784020 | KJ784081 | KJ784142 | KJ784203 | KJ784264 |
| WM 13.346 | KJ783960 | KJ784021 | KJ784082 | KJ784143 | KJ784204 | KJ784265 |
| WM 13.347 | KJ783961 | KJ784022 | KJ784083 | KJ784144 | KJ784205 | KJ784266 |
| WM 13.348 | KJ783962 | KJ784023 | KJ784084 | KJ784145 | KJ784206 | KJ784267 |
| WM 13.349 | KJ783963 | KJ784024 | KJ784085 | KJ784146 | KJ784207 | KJ784268 |
| WM 13.350 | KJ783964 | KJ784025 | KJ784086 | KJ784147 | KJ784208 | KJ784269 |
| WM 13.351 | KJ783965 | KJ784026 | KJ784087 | KJ784148 | KJ784209 | KJ784270 |
| WM 13.352 | KJ783966 | KJ784027 | KJ784088 | KJ784149 | KJ784210 | KJ784271 |
| WM 13.353 | KJ783967 | KJ784028 | KJ784089 | KJ784150 | KJ784211 | KJ784272 |
| WM 13.354 | KJ783968 | KJ784029 | KJ784090 | KJ784151 | KJ784212 | KJ784273 |
| WM 13.355 | KJ783969 | KJ784030 | KJ784091 | KJ784152 | KJ784213 | KJ784274 |
| WM 13.356 | KJ783970 | KJ784031 | KJ784092 | KJ784153 | KJ784214 | KJ784275 |
| WM 13.357 | KJ783971 | KJ784032 | KJ784093 | KJ784154 | KJ784215 | KJ784276 |
| WM 13.358 | KJ783972 | KJ784033 | KJ784094 | KJ784155 | KJ784216 | KJ784277 |
| WM 13.359 | KJ783973 | KJ784034 | KJ784095 | KJ784156 | KJ784217 | KJ784278 |
| WM 13.360 | KJ783974 | KJ784035 | KJ784096 | KJ784157 | KJ784218 | KJ784279 |
| WM 13.361 | KJ783975 | KJ784036 | KJ784097 | KJ784158 | KJ784219 | KJ784280 |
| WM 13.362 | KJ783976 | KJ784037 | KJ784098 | KJ784159 | KJ784220 | KJ784281 |
| WM 13.363 | KJ783977 | KJ784038 | KJ784099 | KJ784160 | KJ784221 | KJ784282 |
| WM 13.379 | KJ783978 | KJ784039 | KJ784100 | KJ784161 | KJ784222 | KJ784283 |
| WM 13.380 | KJ783979 | KJ784040 | KJ784101 | KJ784162 | KJ784223 | KJ784284 |
| WM 13.381 | KJ783980 | KJ784041 | KJ784102 | KJ784163 | KJ784224 | KJ784285 |
| WM 13.382 | KJ783981 | KJ784042 | KJ784103 | KJ784164 | KJ784225 | KJ784286 |
| WM 13.383 | KJ783982 | KJ784043 | KJ784104 | KJ784165 | KJ784226 | KJ784287 |
| WM 13.384 | KJ783983 | KJ784044 | KJ784105 | KJ784166 | KJ784227 | KJ784288 |
| WM 13.385 | KJ783984 | KJ784045 | KJ784106 | KJ784167 | KJ784228 | KJ784289 |
| WM 13.386 | KJ783985 | KJ784046 | KJ784107 | KJ784168 | KJ784229 | KJ784290 |
| WM 13.387 | KJ783986 | KJ784047 | KJ784108 | KJ784169 | KJ784230 | KJ784291 |
| WM 13.388 | KJ783987 | KJ784048 | KJ784109 | KJ784170 | KJ784231 | KJ784292 |
| WM 13.389 | KJ783988 | KJ784049 | KJ784110 | KJ784171 | KJ784232 | KJ784293 |
| WM 13.390 | KJ783989 | KJ784050 | KJ784111 | KJ784172 | KJ784233 | KJ784294 |
| WM 13.391 | KJ783990 | KJ784051 | KJ784112 | KJ784173 | KJ784234 | KJ784295 |
| WM 13.392 | KJ783991 | KJ784052 | KJ784113 | KJ784174 | KJ784235 | KJ784296 |
| WM 13.393 | KJ783992 | KJ784053 | KJ784114 | KJ784175 | KJ784236 | KJ784297 |
| WM 13.394 | KJ783993 | KJ784054 | KJ784115 | KJ784176 | KJ784237 | KJ784298 |
